# The crystal structure of iC3b-CR3 αI reveals a modular recognition of the main opsonin iC3b by the CR3 integrin receptor

**DOI:** 10.1038/s41467-022-29580-2

**Published:** 2022-04-12

**Authors:** Francisco J. Fernández, Jorge Santos-López, Rubén Martínez-Barricarte, Javier Querol-García, Héctor Martín-Merinero, Sergio Navas-Yuste, Martin Savko, William E. Shepard, Santiago Rodríguez de Córdoba, M. Cristina Vega

**Affiliations:** 1 Centro de Investigaciones Biológicas Margarita Salas, Agencia Estatal Consejo Superior de Investigaciones Científicas, 28040 Madrid, Spain; 2 Abvance Biotech srl, 28003 Madrid, Spain; 3 Centro de Investigación Biomédica en Enfermedades Raras, 28040 Madrid, Spain; 4 Synchrotron SOLEIL, L’Orme des Merisiers Saint-Aubin, 91192 Gif-sur-Yvette, France; 5 Present Address: Division of Genetic Medicine, Department of Medicine, Vanderbilt University Medical Center, Nashville, TN 37232 USA

**Keywords:** Complement cascade, X-ray crystallography

## Abstract

Complement activation on cell surfaces leads to the massive deposition of C3b, iC3b, and C3dg, the main complement opsonins. Recognition of iC3b by complement receptor type 3 (CR3) fosters pathogen opsonophagocytosis by macrophages and the stimulation of adaptive immunity by complement-opsonized antigens. Here, we present the crystallographic structure of the complex between human iC3b and the von Willebrand A inserted domain of the α chain of CR3 (αI). The crystal contains two composite interfaces for CR3 αI, encompassing distinct sets of contiguous macroglobulin (MG) domains on the C3c moiety, MG1-MG2 and MG6-MG7 domains. These composite binding sites define two iC3b-CR3 αI complexes characterized by specific rearrangements of the two semi-independent modules, C3c moiety and TED domain. Furthermore, we show the structure of iC3b in a physiologically-relevant extended conformation. Based on previously available data and novel insights reported herein, we propose an integrative model that reconciles conflicting facts about iC3b structure and function and explains the molecular basis for iC3b selective recognition by CR3 on opsonized surfaces.

## Abstract (web summary)

Complement activation on foreign cell surfaces leads to the generation of complement opsonins, which activate complement receptor type 3 (CR3) and pathogen clearance by macrophages. Here, the authors reveal structural basis of the interaction between human opsonin iC3b and the von Willebrand A inserted domain of the α chain of CR3.

## Introduction

The complement system comprises >30 soluble and membrane-associated protein factors that survey the blood and interstitial fluids for the presence of viruses, bacteria, eukaryotic pathogens, apoptotic cell debris, and immune complexes^1,2^. Complement activation on target surfaces drives the proteolytic activation of complement factor 3 (C3) to C3b, which becomes covalently attached to surface-exposed nucleophiles via a transacylation mediated by its reactive internal thioester bond. C3b is then further processed by factor H and factor I to generate iC3b, the main opsonin and yet the least well-understood proteolytic product of C3 both in function and structure. Eventually, iC3b is cleaved a second time by factor I to yield surface anchored C3dg and free C3c^3,4^. Communication between the innate and adaptive immunity mediated by the recognition of these C3-derived opsonins by complement receptors is essential for mounting and orchestrating an efficient response against pathogens and for the maintenance of homeostasis^5–9^.

Surfaces opsonized with iC3b are recognized by the homologous leukocyte-specific β_2_-integrin complement receptors type 3 (CR3, also known as CD11b/CD18, Mac-1 or integrin α_M_β_2_) and type 4 (CR4, also known as CD11c/CD18 or integrin α_X_β_2_). CR3 and CR4 are heterodimeric type I membrane proteins, each consisting of a common β chain (β_2_) and a unique α chain (α_M_ or α_X_). The N-terminal end of the α chain contains the iC3b-binding von Willebrand type A (VWA) domain or α chain inserted domain (αI), a specialized region characterized by a modified Rossman-fold architecture and a metal ion-dependent adhesion site (MIDAS) motif^10^. Both integrin receptors display poor affinity for C3b but high affinity for iC3b, their cognate ligand. CR3 is present on myeloid cells, including neutrophil granulocytes, monocytes, macrophages, and activated lymphocytes, and lymphoid natural killer cells^11^. It mediates immune adhesion-dependent processes such as adhesion to endothelium, phagocytosis of opsonized foreign particles, and other activation events that promote the innate and the adaptive branches of the immune system. Genetic defects impairing leukocyte immune adhesion cause life-threatening diseases; for example, mutations in the *ITGB2* gene encoding the common β chain cause the autosomal recessive disease leukocyte adhesion deficiency type-I (LAD-I) characterized by recurring bacterial infections and poor wound healing^12,13^, and CR3 deficiency alters susceptibility to immune complexes^14^.

Over the last decade, several incremental advances have been made in our understanding of the structure and function of iC3b. Factor I-mediated conversion of C3b to iC3b involves the proteolytic release of the C3f fragment (residues 1282–1298), a process that disrupts the integrity of the CUB domain connecting the core of iC3b (so-called C3c moiety) to the thioester-containing domain (TED)^15–17^. As a result, the TED domain could shift its position and orientation as observed in C3b toward a new stable arrangement or remain free-ranging at the end of a flexible tether (the CUB remnants), as suggested by negative stain electron microscopy^18,19^. However, the lack of a high-resolution structure of iC3b has hampered efforts to understand the biology of this critical opsonin. In addition, quantitative binding studies by surface plasmon resonance (SPR) of surface-immobilized C3 derivatives with CR3 αI as analyte have suggested a heterogeneous interaction involving multiple interfaces with affinities ranging from low m*M* to high n*M*^10^.

More recently, research efforts have begun to decipher the molecular and structural basis underlying iC3b recognition by CR3 and CR4. The structure of a complex between the TED domain and the CR3 αI domain (PDB 4M76) revealed an interface centered at the Ni^2+^ cation present in the MIDAS motif of αI in the crystal structure^10^. Despite considerable sequence identity (60% overall, 47% within the αI domain), CR3 and CR4 appear to utilize different nonoverlapping binding sites on iC3b^20^. Electron micrographs of iC3b and the near full-length ectodomain of CR3 (the CR3 headpiece) revealed complexes containing predominantly the same TED-αI domain interaction. By contrast, electron micrographs of iC3b complexed with the CR4 αI domain showed no interactions with the TED domain while instead providing evidence for up to two independent CR4 αI binding sites on iC3b. The dominant binding site, present in all complexes, was located next to the macroglobulin (MG) domains MG3–MG4, while a less frequently used binding site was found in the vicinity of the C345c domain^20^.

In this work, we have revisited the structure of iC3b in the context of its interaction with the CR3 αI domain. By solving the crystal structure of the iC3b–CR3 αI complex at 3.4-Å resolution, we show that, in addition to the known interaction surface of CR3 with the TED domain, there are two more CR3 αI binding sites on the C3c moiety of iC3b. These interfaces define iC3b–CR3 αI complexes that differ in the location and orientation of the TED domain and, therefore, in the orientation of the C3c moiety in relation to the surface of the opsonized particle. The resulting iC3b conformations, which we have termed upright (iC3b^U^) and upside down (iC3b^D^), are compatible with binding the much larger CR3 headpiece. Combining crystallographic, solution small-angle X-ray scattering (SAXS), and interaction data, we have built an integrative model to inform research into the dynamics of the iC3b–CR3 complex during molecular recognition processes of opsonized surfaces containing both iC3b and C3dg molecules.

## Results

### Crystal structure of iC3b–CR3 αI domain

**Fig. 1:**
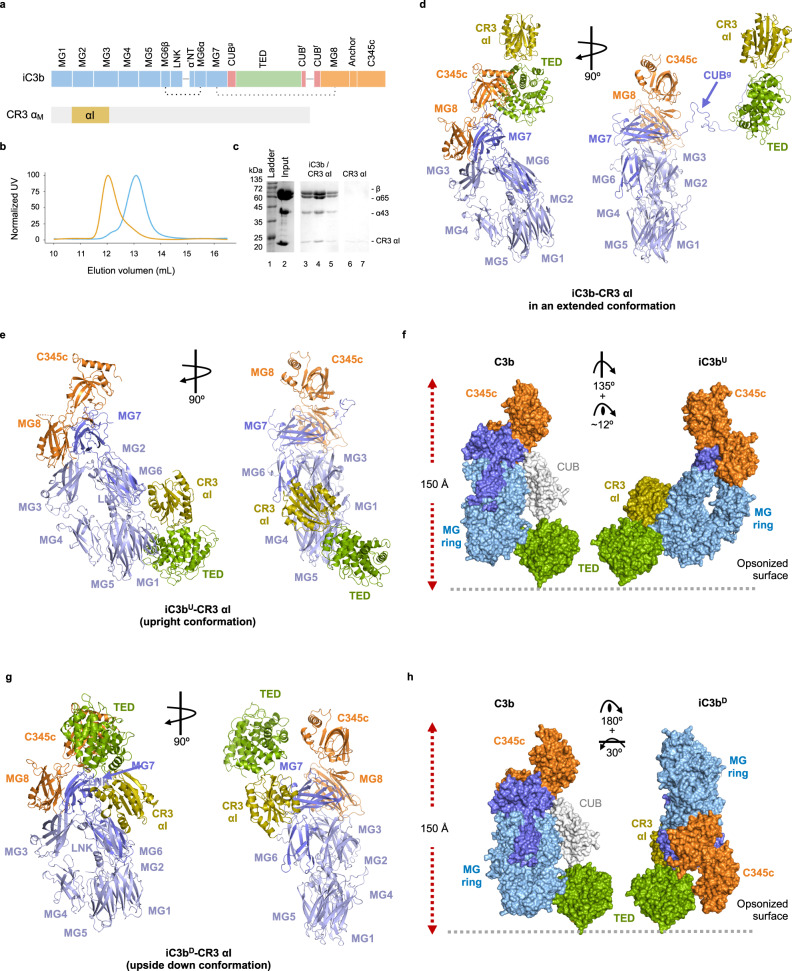
Crystal structure of iC3b–CR3 αI. **a** Schematic representation of the domain architecture of iC3b and the von Willebrand A (VWA) or inserted (I) domain of CR3 (CR3 αI). **b** Analytical SEC (anaSEC) traces of iC3b–CR3 αI (orange) and iC3b (blue). **c** Coomassie-stained SDS-PAGE electrophoresis of peak fractions for the iC3b–CR3 αI chromatogram shown in (**b**). We repeated at least five independent purifications of the iC3b–CR3 αI complex with similar results. **d** Ribbon representation of the extended conformation of the iC3b–CR3 αI complex. **e** Ribbon representation of the iC3b^U^–CR3 αI complex shown in two orientations related by a 90° rotation around a vertical axis on the plane of the figure. **f** Comparison of C3b (left) with iC3b^U^ (upright conformation) with respect to a hypothetical opsonized surface covalently anchored to the TED domain via Gln991. C3b and iC3b^U^ are shown as molecular surfaces, and the CUB domain of C3b is shown in white. The orientation of the TED domain in C3b and iC3b is the same. The rotations necessary to superimpose C3b into iC3b^U^ are indicated. **g** Ribbon structure of the iC3b^D^–CR3 αI complex. **h** As in g for iC3b^D^ (upside-down conformation). The same color scheme is applied to all panels: the β-chain is shown in light blue, the α65 chain in slate blue (except the TED domain, which is shown in green), the α43 chain in orange, and the CR3 αI domain in olive. Complexes in panels (**d**–**f**) are shown in the same orientation for clarity’s sake. Source data are provided as a Source Data file.

We have determined the crystallographic structure of the heterodimeric iC3b–CR3 αI domain complex at 3.4-Å resolution from crystals grown from reconstituted stoichiometric protein complex purified by size-exclusion chromatography (Fig. 1a–c, Supplementary Table 1, and Supplementary Fig. 1). The final model was refined to convergence using noncrystallographic symmetry (NCS) restraints and model restraints derived from the structures of the isolated subunits. The asymmetric unit contains two copies of iC3b–CR3 αI; one is an iC3b-derived C3c subunit, presumably generated by the spontaneous chemical cleavage of iC3b (Fig. 1 and Supplementary Fig. 2). Unbiased electron density maps show that the CUB^g^ region (residues 913–954), which connects the TED domain to the MG ring via the MG7 domain, has adopted an extended conformation (~62 Å measured along the peptide chain, 2.4 times longer than the same region in C3) tethering the C3c moiety of iC3b to the TED–CR3 αI subcomplex in a crystal neighbor molecule (Fig. 1d and Supplementary Fig. 3). These data support that the CUB^g^, CUB^f1^ (residues 1269–1281), and CUB^f2^ (residues 1299–1330) segments making up the CUB domain in C3 and C3b are all present in the crystal but do not remain associated in a well-folded domain (Fig. Supplementary 3). Of these segments, only CUB^g^ could be traced in the crystal structure, while CUB^f1^ and CUB^f2^ were disordered. Interestingly, the CUB^g^ adopted an extended configuration reminiscent of natively disordered proteins, as previously inferred from hydrogen/deuterium exchange and other functional experiments^17^ (Supplementary Fig. 3). Since the length across the CUB domain is essentially unchanged from C3 to C3b (26 to 29 Å), it is the increased length and flexibility of the CUB^g^ region in iC3b that allows the relocation of the TED domain to a position ~50–72 Å away from the MG ring (Supplementary Fig. 4).

The two C3c moieties in the asymmetric unit show no significant differences and can be superimposed with a root-mean-square displacement (RMSD) of 0.76 Å (1113 residues) (Supplementary Fig. 5). They twist around one another in an antiparallel fashion that creates contact surface areas between opposite C3c moieties. Thus, the C345c domain (residues 1518–1661) of each C3c subunit interacts with the two extremes of the MG ring, the MG1 (residues 1–103) and LNK (582–642) domains, on the opposite C3c subunit; and the MG2 and MG7 domains (residues 104–206 and 807–912, respectively) of one C3c subunit interact with the same domains on the opposite C3c moiety after rotation by ~90°. Furthermore, the planes defined by the C3c moieties cross at an ~65° angle, creating a long (~100 Å), deep (~15–25 Å) crevice on one side; toward the opposite side, they form a far broader and shallower depression characterized by the complementary tilt (~115°).

The iC3b–CR3 αI complex crystal presents two distinct interaction surfaces between the TED-bound CR3 αI subcomplex and the C3c MG ring from both iC3b molecules. In the extended conformation of iC3b, the TED-αI subcomplex occupies a position that is further stabilized by the lateral interaction of the same αI domain with the MG ring of a crystallographic neighboring C3c moiety situated at one end of the shallow depression between two adjacent C3c MG rings (Fig. 1d). Interestingly, a crystallographically equivalent TED-αI subcomplex can occupy an almost symmetric environment at the opposite end of the same depression. The first C3c-αI interface straddles the MG1–MG2 domains (Fig. 1e–f), whereas the second C3c-αI interface contacts the MG6–MG7 domains (Fig. 1g–h). The presence of three distinct iC3b-binding surfaces (one involving the TED domain, two involving the C3c moiety) on this small ~185-amino-acid domain is remarkable.

To further decipher the nature and structural consequences of these C3c-αI interfaces, we partitioned them into two different iC3b–CR3 αI interactions depending on the specific site of attachment for CR3 αI on the C3c moiety and the corresponding orientation of the TED–CR3 αI subcomplex. Although iC3b–CR3 αI complexes involving the C3c moiety do not appear to be sufficiently stable to play significant roles at the low to moderate concentrations typical of biochemical assays and electron microscopy, they might be important in high-density clusters like those present on opsonized surfaces. Therefore, we referred to these complexes as iC3b^U^–CR3 αI and iC3b^D^–CR3 αI (Fig. 1e–h). The known 1:1 stoichiometry for the iC3b–CR3 αI complex and the features of the negative stain electron microscopy studies carried out on the iC3b–CR3 headpiece^20^ support this assignment (see below). In iC3b^U^–CR3 αI, which retains an upright conformation for the C3c moiety in relation to the TED domain and therefore with respect to the opsonized surface, CR3 αI is bound to the MG1–MG2 domains of iC3b (Fig. 1e, see below). In contrast, C3b^D^–CR3 αI exhibits an upside-down conformation of the C3c moiety with respect to the orientation of the TED domain and is characterized by a more compact structure where CR3 αI binds to the MG6–MG7 surface of iC3b near the C345c domain (Fig. 1g).

**Fig. 2:**
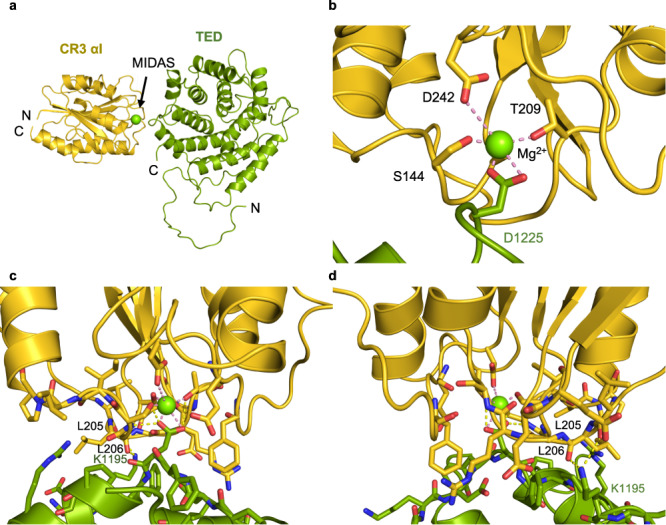
Interface of the TED–CR3 αI subcomplex of iC3b–CR3 αI. **a** Overall structure of the subcomplex. Cartoon representations of the TED domain and part of the CUB^g^ segment are colored in splitpea and the αI domain of CR3 in gold. The Mg^2+^ cation of the MIDAS motif is shown as a green sphere. **b** Zoom into the Mg^2+^ coordination sphere. Distances between oxygen atoms and the Mg^2+^ ion are shown as wheat dashed lines. **c**, **d** Two side views into the TED-αI interface related by an almost 180° rotation around a vertical axis. In addition to the coordination bonds, interchain hydrogen bonds are shown as dashed lines (colored in yellow).

The TED–CR3 αI domain interaction identified in the crystal structure largely recapitulates the interaction previously observed for the isolated subunits in the presence of Ni^2+^ (PDB 4M76)^10^ (Fig. 2a). Overall, the RMSD between the TED–CR3 αI domain subcomplex in iC3b or in isolation is 0.55 Å (Supplementary Fig. 6). The present TED–CR3 αI complex contains a physiological Mg^2+^ cation bound to the MIDAS motif (Fig. 2b). Furthermore, the C-terminal helix α10 of CR3 αI (residues 303–313), partially disordered in the previous TED–CR3 αI structure, is fully ordered in electron density. The TED–CR3 αI interface is electrostatically driven, with ten charge-assisted hydrogen bonds and a direct MIDAS Mg^2+^-carboxylate interaction (Asp1225^iC3b^) concentrated across a small surface area (466 Å^2^) (Fig. 2c–d, Supplementary Table 2). The CR3 αI interfacial residues are contributed by several noncontiguous amino acid segments (residues 142–147 from helix α1, 178–179 between the β4-α8 connection, 205–209 between helices α3-α4, 244–246 between β3-α6, and 276 between β4-α8) while most of the TED residues engaged in the interaction cluster along two adjacent and tightly kinked helices (residues 1221–1232, with the kink at Asp1225) (Fig. 2c–d).

### CR3 αI binding site on iC3b^U^ is mediated by MG1–MG2

**Fig. 3:**
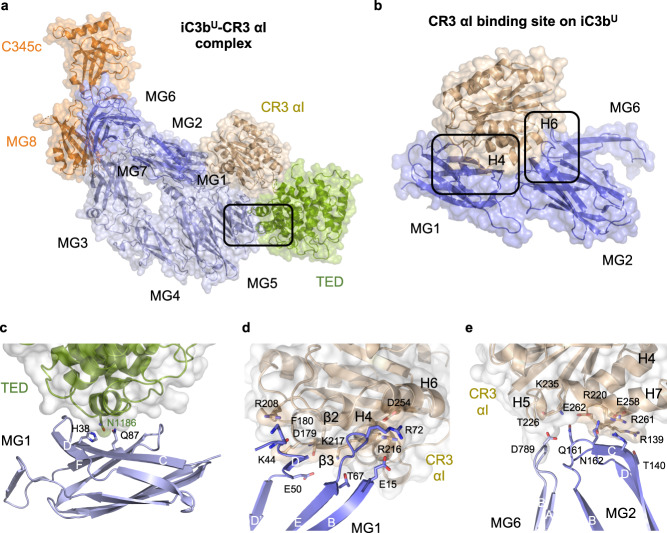
CR3 αI binding site and interactions with the C3c moiety and the TED domain in iC3b^U^–CR3 αI. **a** Overall structure of the iC3b^U^–CR3 αI complex in the upright orientation characteristic of the conformation. Structures are shown in cartoon representation and overlaid by a semi-transparent molecular surface. iC3b^U^ is color-coded as in Fig. 1. CR3 αI is shown in wheat color. The region inside the black outline is shown in (**c**). **b** Interaction of CR3 αI with the MG domains MG1–MG2. The boxed areas are magnified in panels (**d**) and (**e**). **c** Interface between the TED and MG1 domains. **d** Close-up of the interaction of CR3 αI with the MG1 domain. **e** Close-up of the interaction of CR3 αI with the MG2 domain. The tip of the turn between β-strands βA and βB of MG6 provides an additional contact. Amino acid residues which establish hydrogen bond or salt bridge interactions across the interfaces shown in panels (**c**–**d**) are shown as sticks.

To adopt the iC3b^U^–CR3 αI conformation (U stands for upright), the C3c moiety should rotate from the original position in C3b by ~135° around a longitudinal axis parallel to its longest dimension, and then tilt by ~12° (Figs. 1h, 3a). In its final position in iC3b^U^, the C3c moiety would have undergone a ~26-Å translation with respect to the TED domain, which places the latter domain on the opposite side of the MG ring that it occupied in C3b. As a result, the overall shape of the iC3b^U^–CR3 αI is more elongated (by ~15 Å) than that of iC3b^D^–CR3 αI (Figs. 1e–h, 3a). Following this rearrangement, the binding site for CR3 αI on the TED domain, partially occluded in C3b, becomes accessible in iC3b (Fig. 3a–c).

The binding site for the αI domain of CR3 on the C3c moiety of iC3b^U^ encompasses MG1 (residues 15 and 43–71), MG2 (residues 138–190), and a single residue from MG6 (Asp789) that lies near the lower half of the MG2 domain (Fig. 3b,d–e and Supplementary Table 3). In CR3 αI, the residues involved in contacting the MG1–MG2 region of iC3b span two distinct segments (residues 178–181 and 208–264) (Fig. 3d,e and Supplementary Table 3). The interaction area buried at the interface involving the C3c moiety is larger than the area buried by the interaction of CR3 αI with the TED domain (874 Å^2^ vs. 466 Å^2^), and it contains mostly polar and charged interactions with eight hydrogen bonds and five salt bridges. In iC3b^U^–CR3 αI, the TED domain forms a small but stable interaction surface (382 Å^2^) with the MG1 domain via contacts made between an α-helix of the TED domain (H11, residues 1183–1191) and residues on the outward-facing β-sheet of MG1 (Fig. 3c and Supplementary Table 3).

An interesting feature of iC3b^U^–CR3 αI is the involvement of glycosylated sites on both interacting surfaces. Asn63 in MG1 of iC3b is known to be glycosylated, as shown in all crystal structures derived from C3 purified from plasma with intact glycosylation (Supplementary Fig. 8). Accordingly, in the iC3b–CR3 αI crystal structure, the core *N*-acetyl-glucosamine (NAG) disaccharide linked to an α(1–4)-mannopyranoside residue could be modeled in electron density. Reciprocally, on CR3 αI, Asn224, a residue directly facing the groove across MG2 and MG6 and, therefore, part of the interaction surface with iC3b, is known to be glycosylated. Since the αI domain used for crystallization was expressed in bacteria, it lacks glycosylation. In the native complex with CR3, though, the two glycosylated residues (Asn63^iC3b^ and Asn224^CR3^) would stand at opposite sides of the cleft ~21 Å apart. The glycan moiety on Asn224^CR3^ would likely be able to make additional contacts across the polar interface with iC3b. In the cleft between MG1–MG2, residues from three different MG domains come together so that they could potentially interact with the glycosylated side chain of Asn224^CR3^: Asp17 (MG1), Lys133 (MG2), Asp532 (MG6α), and Lys790 (MG6β).

### CR3 αI binding to iC3b^D^ αI is mediated by MG6–MG7

**Fig. 4:**
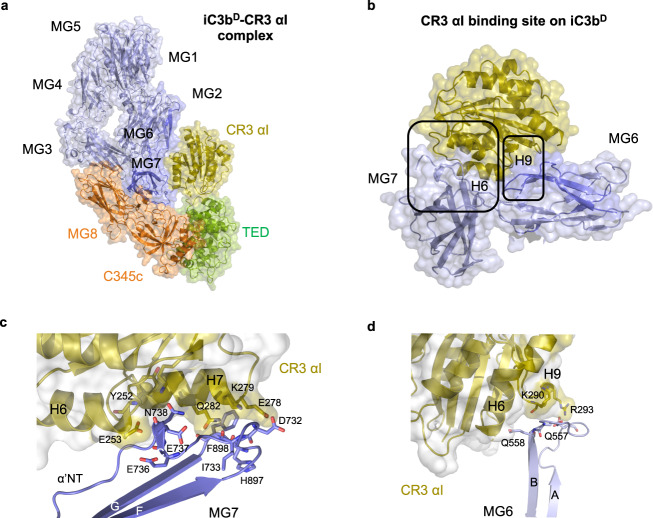
CR3 αI binding site and interactions with the C3c moiety and the TED domain in iC3b^D^–CR3 αI. **a** Overall structure of the iC3b^D^–CR3 αI complex in the upside-down orientation characteristic of the conformation. Structures are shown in cartoon representation and overlaid by a semi-transparent molecular surface. iC3b^D^ is color-coded as in Fig. 1. CR3 αI is shown in olive color. **b** Interaction of CR3 αI with the MG domains MG6–MG7. The boxed areas are magnified in panels **c** and **d**. (**c**) Detail of the interaction of CR3 αI with the α’NT motif and MG7 domain. **d** Close-up of the interaction of CR3 αI with the MG6 domain. Amino acid residues which establish hydrogen bond or salt bridge interactions across the interfaces shown in panels c–d are shown as sticks.

In the iC3b^D^–CR3 αI subcomplex (D stands for upside-down conformation), the αI domain binds to a surface that straddles the α’NT, MG6, and MG7 domains on the upper edge of the MG ring of iC3b^D^ (Figs. 1g–h, 4). Considering that the TED domain is covalently attached to surfaces via its reactive thioester-mediated covalent bond, the conformational change from C3b to iC3b^D^ requires the rotation of the C3c moiety of iC3b^D^ by 180°, which ultimately turns the C3c moiety upside down with respect to the upright pose in C3b^U^ (Fig. 4a). Additional minor rotational adjustments (~15–30°) around the other two perpendicular directions are sufficient to ensure a near-perfect alignment (normal to the opsonized surface) for iC3b^D^.

The buried surface area in iC3b^D^–CR3 αI is slightly (4%) smaller than in iC3b^U^–CR3 αI (840 Å^2^ vs. 874 Å^2^). Although the buried surface area is larger than the TED-αI interaction (466 Å^2^), it contains slightly fewer polar interactions. The αI domain-contacting residues in iC3b^D^ are provided by the 3_10_ helix in α’NT (residues 731–744) preceding MG6 as it runs down the long axis of the MG7 domain, onto which it rests, as well as from β-strands A-B belonging to MG6 (residues 547–558 and 770–772) and MG7 (residues 896–904), and residue Gln805 at the sharp kink responsible for the right-angle orientation between MG6 and MG7 (Fig. 4b–d and Supplementary Table 4). The involvement of residues 731–744 of the α’NT segment in stabilizing this interface could provide the structural underpinning for previously published mutagenesis data. Indeed, the joint mutation of Glu736-Glu737 in an EEN motif to their isosteric amide Gln to form a QQN motif, a change which could produce structural alterations near the complex interface, has been demonstrated to result in a substantial reduction in the interaction between iC3b and CR3^21^. On the CR3 αI domain, the interaction surface is defined by residues mostly found on two long helices (α6 and α7) that penetrate the groove between the MG6–MG7 interface (residues 234–290) (Fig. 4b,d). In this extended interface, three salt bridges occur in a narrow surface patch formed by residues grafted from the end of helix α9 in αI (Lys290 and Arg293) and the short turn between β-strands A-B in MG6 (Gln557 and Gln558) (Fig. 4d and Supplementary Table 4).

The orientation of the TED–CR3 αI subcomplex in relation to the C3c moiety of iC3b has changed in iC3b^D^–CR3 αI compared to iC3b^U^–CR3 αI (Fig. 4a). In the upside-down conformation (iC3b^D^), the TED domain lies on the opposite side of the MG ring compared with C3b (PDB 2I07)^15^ or C3 (PDB 2A73)^22^ (Fig. 4a and Supplementary Fig. 4). This location for the TED domain must have been rendered possible by the partial unfolding and extension of the CUB domain present in the iC3b molecule. The structure of C3b can be superimposed onto iC3b (RMSD 1.33 Å over 1104 Cα atoms) with minor differences in their shared domains with the obvious exception of the position occupied by the TED domain. The clearest difference concerns the position of the C345c domain, which has rotated slightly, causing the three external helices of the domain to show a maximum displacement of 4.0 Å (Supplementary Fig. 7).

### Both iC3b–CR3 αI complexes are compatible with full CR3 binding

The identification of two distinct iC3b–CR3 αI interaction surfaces involving different iC3b conformations in the crystallographic structure (Fig. 1e, g) prompted us to evaluate the compatibility of the reconstructed structures of iC3b^D^–CR3 αI and iC3b^U^–CR3 αI with binding the much larger CR3 ectodomain. Although the structure for the headpiece of CR3 remains unsolved except for the αI domain, there are several structures available for the other homologous integrin receptors which share the same β_2_ chain present in CR3: CR4 and lymphocyte function-associated antigen-1 (LFA-1, also known as CD11a/CD18 or integrin α_L_β_2_). Moreover, pairwise sequence identity between the α chains of CR3 and CR4 or LFA-1 are 60% and 31%, respectively, exceeding the threshold (30% identity) established by template-based modeling, based on the observation that protein–protein complexes usually interact in the same way if their interacting pairs share >30% sequence identity^23^. The structures of the upper part of the CR4 (PDB 4NEH)^24^ and LFA-1 (PDB 5E6S)^25^ headpiece can be superimposed by rigid-body superposition (RMSD 2.2 Å over 391 out of 579 Cα atoms), except for the αI domains, which are rotated by ~90°. Using a flexible superposition algorithm^26^ achieves an excellent superposition (RMSD 0.99 Å over 546 out of 579 Cα atoms) after decomposition of the α chain in three rigid bodies, the C-terminal β-propeller domain, helix α1 (residues 142–162 in CR4 αI), which is inserted between the β1 and β2 strands of the αI domain, and the rest of the αI domain. These observations indicate not only that the upper part of the CR4 and LFA-1 headpieces is greatly conserved at the level of structure, but also that the αI domain of integrin receptors can freely rotate and tilt around the platform formed by the α-chain β-propeller and β-chain βI domains of the headpiece^25^.

**Fig. 5:**
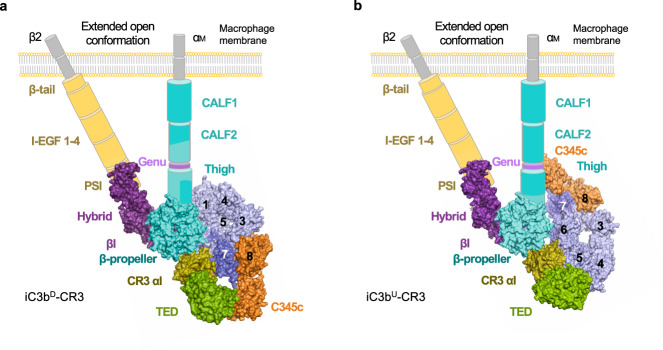
Molecular architecture of the iC3b–CR3 complex. Multi-scale model of the interaction of iC3b with full CR3 in the upright iC3b^U^ conformation (**a**) or the upside-down iC3b^D^ conformation (**b**). Model building is explained in the main text and the Supplementary Information. Domains of CR3 α_M_ and β_2_ chains with known atomic structures used for model building are shown as molecular surfaces, while the remaining domains are shown in schematic form. The angle between the hybrid and the βI domains characteristic of the extended open conformation have been modeled approximately from existing structural evidence.

Homology models for the iC3b–CR3 headpiece can be constructed by overlaying the upper part of CR4 (PDB 4NEH) or LFA-1 (PDB 5E6S) headpiece, consisting of the α-chain αI and β-propeller domains and the β-chain βI and hybrid domains, onto the structures of iC3b^D^–CR3 αI and iC3b^U^–CR3 αI by superposition of their corresponding αI domains (Fig. 5 and Supplementary Fig. 9). The three αI domains can be accurately superimposed (RMSD 1.1–1.6 Å over >90% Cα atoms), and the β-strands at the hinge region connecting the αI with the β-propeller domains of the α_Μ_ chain of CR3 are correctly aligned. Since the CR4 and LFA-1 structures used as templates for the CR3 headpiece were solved in the unliganded state, and the αI domain is known to allow a high degree of flexibility around its connection to the remainder of the headpiece, some adjustment is expected to be required to relieve structural clashes in the homology model of a complex with iC3b. The architecture of the iC3b^U^–CR3 αI complex is easily compatible with binding to the CR3 headpiece since homology models based on CR4 or LFA-1 reveal clash-free superpositions even without adjustments for the position or orientation of the α-chain β-propeller domain or the β-chain βI domain (Fig. 5a). Inspection of the models based on iC3b^D^–CR3 αI immediately reveals that a slight rotation of the β-propeller domain about the fixed αI domain is enough to yield a clash-free complex (Fig. 5b). Interestingly, the pseudo-symmetric arrangement of the two CR3 αI binding sites leads to homology models whereby the β-chain βI domain (a von Willebrand domain as αI) ends up being placed near the unoccupied CR3 αI site. Thus, in the homology model for iC3b–CR3 based on iC3b^U^–CR3 αI, βI becomes approximately aligned with the MG6–MG7 domains of iC3b. In contrast, in the homology model based on iC3b^D^–CR3 αI, βI ends up near the MG1–MG2 interface.

### iC3b–CR3 αI complex in solution

**Fig. 6:**
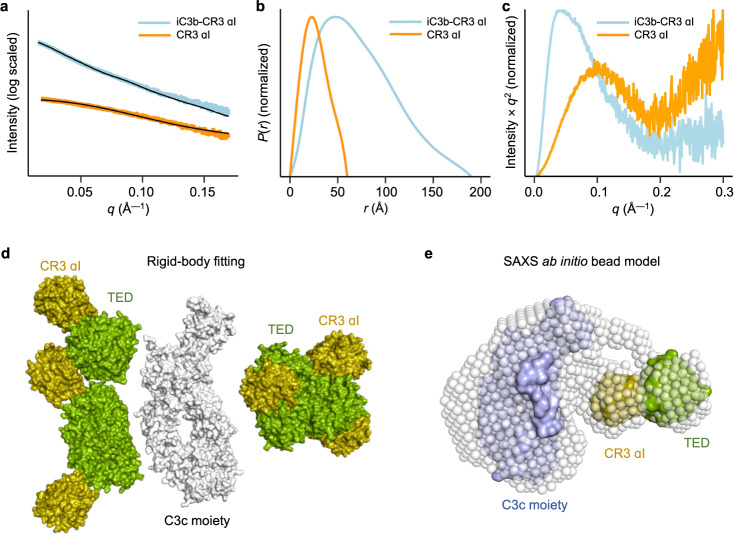
SAXS shape restoration of the iC3b–CR3 αI complex. **a** Experimental scattering curves for iC3b–CR3 αI (cyan), the same sample used for crystallization, and the αI domain (orange). **b** Pair distance distribution function *P*(*r*) plotted against distance (*r*). **c** Kratky plot showing that the iC3b–CR3 αI complex (cyan) is a compact, well-folded structure. In contrast, the structure of the constitutively opened mutant CR3 αI domain is less compact as the curve trends upward as the momentum transfer *q* increases. **d** Composite model showing the molecular surface for the C3c moiety of iC3b (in white) surrounded by the best-fitting (*χ*^2^ = 2.0–8.0) TED–CR3 αI domain subcomplexes (shown in molecular surface representation, the TED domain in green and the CR3 αI domain in olive). The labels indicate the position of the subunits in the TED–CR3 αI domain subcomplexes. **e** Representative bead model calculated for the iC3b–CR3 αI complex from the SAXS scattering data ab initio with *DAMMIF/N*. The crystallographic C3c moiety of iC3b (in light blue) and the TED–CR3 αI domain subcomplex (TED in green and the CR3 αI domain in olive) were fitted by rigid-body modeling. Source data are provided as a Source Data file.

SAXS scattering data for the iC3b–CR3 αI complex and the free CR3 αI domain were collected to assess the shape and dynamic properties and to control for the presence of unbound αI contaminating the iC3b–CR3 αI domain sample. Supplementary Table 5 summarizes the SAXS results. Analysis of the SAXS data indicates that the iC3b–CR3 αI domain complex has a flexible though spatially constrained shape (Fig. 6a–c). The concentration-independent molecular mass estimates of the iC3b–CR3 αI complex (194.4 ± 34.5 kDa) lie within the theoretical mass range for iC3b–CR3 αI (195.4 kDa, assuming 173.2 kDa for iC3b and 22.2 kDa for CR3 αI). The shape classification of the data and the *P*(*r*) vs. *r* profile show that the iC3b–CR3 αI complex has a relatively compact shape in solution with an *R*_g_ of ~55 Å (not much greater than those of C3, C3b, or iC3b) and a *D*_max_ of ~192.5 Å (slightly larger than that of C3b) (Fig. 6b and Supplementary Table 5)^19^.

The inherent flexibility of the CUB remnants in iC3b gives rise in solution to various conformations with different relative orientations and distances between the C3c moiety and the TED domain^18,19^. In agreement with this flexibility, the SAXS data for the iC3b–CR3 αI complex could be approximated by the theoretical scattering of the structure of iC3b^U^–CR3 αI complex (*χ*^2^ = 4.3) and, though worse, by that of the iC3b^D^–CR3 αI complex (*χ*^2^ = 8.5). To investigate the range of conformations present in solution, we carried out multiple rounds (50) of rigid-body modeling of the iC3b–CR3 αI domain complex with *SASREF* using different configurations as starting points for the simulation and allowing the C3c moiety and the TED–CR3 αI subcomplex to move independently as rigid bodies. These starting configurations included the looser arrangement of iC3b in the extended conformation and the compact conformations iC3b^D^–CR3 αI and iC3b^U^–CR3 αI complexes (Fig. 1). By assessing the outcome of multiple rounds of rigid-body fitting, we could cluster the more likely locations for the TED–CR3 αI subcomplex in solution with *χ*^2^ values as low as 2.0 (Fig. 6d). The nonrandom placement of the TED–CR3 αI subcomplex with respect to the MG ring in solution may be interpreted as defining highly probable loci between which the TED–CR3 αI subcomplex could swing by rotation and translation. The family of solutions obtained comprises arrangements that resemble the extended conformation with independent modules connected via the CUB remnants (Figs. 6d, 1d) and other solutions that loosely approximate the compact complexes observed in iC3b^D^–CR3 αI and iC3b^U^–CR3 αI (Figs. 6e and 1e, g).

In addition, SAXS-based ab initio shape restoration was performed to gain further insight into the organization and behavior of the complex in solution. The consensus refined low-resolution (~42 ± 3 Å by *SASRES*) bead model consists of two distinctive modules. The largest module or core of the bead model corresponds by size and shape to the MG ring and adjacent domains, whereas the smallest volume, which is slightly detached from the core, can accommodate the TED–CR3 αI subcomplex snugly. A representative SAXS-derived bead model with the C3c moiety and the TED–CR3 αI subcomplex fitted as rigid bodies is shown in Fig. 6e. The complete range of shape variation is illustrated in Supplementary Fig. 10. Since the TED–CR3 αI subcomplex appears to be sticking out of the iC3b MG ring in most bead models, the SAXS-based model in solution argues for a significant amount of flexibility in how the TED–CR3 αI subcomplex orients itself with respect to the MG ring.

In summary, SAXS data (Fig. 6) and size-exclusion chromatography (Fig. 1b–c) show that the complex is a 1:1 heterocomplex in solution at the assayed concentrations that is flexible enough around the CUB tether to adopt a continuum of conformations, most of which are extended with little direct contact between the C3c moiety and the TED domain (Fig. 6d, e). This situation is consistent with the extended conformation observed in the crystallographic structure (Fig. 1d) and previous negative stain electron microscopy studies^18–20^, and it agrees with the heterogeneous binding pattern observed by SPR for αI binding to surface-immobilized iC3b^10^.

### Surface interaction between iC3b or C3c and the CR3 αI domain

Surface plasmon resonance (SPR) has been extensively used to probe the interaction between complement factors and their receptors^10,19^. A perceived advantage of these studies is that binding occurs on a biocompatible surface, thus mimicking the natural environment characterized by the covalent attachment of the C3b, iC3b, or C3dg opsonins to biological surfaces. Previous studies have shown that surface-immobilized C3b, iC3b, C3c, and C3d interact with the CR3 αI domain, although with markedly different kinetics and affinity constants^10^. Except for C3d, binding to the CR3 αI domain could only be described entirely in terms of a heterogeneous binding model^10^. The MG ring-containing C3b, iC3b, and C3c exhibited weak equilibrium dissociation constants for CR3 αI (*K*_D_ ~ 10^−4^ to 10^−3^ *M*). This low-affinity binding mode has been described as quantitatively equivalent to the binding of acidic side chains^27^. The strongest affinity constants were measured for iC3b and C3d (*K*_D_ ~ 10^−7^ to 10^−6^ *M*).

**Fig. 7:**
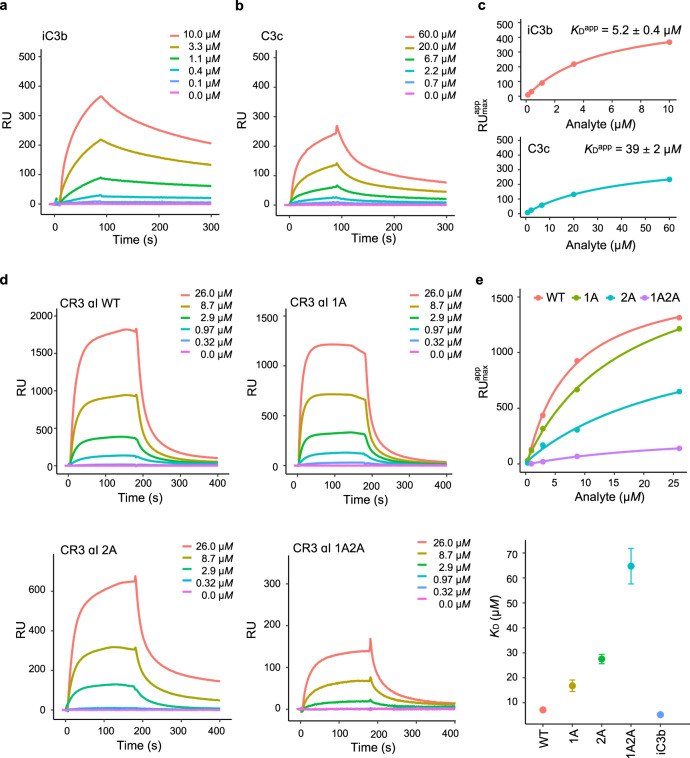
SPR analysis of the interaction between the CR3 αI domain and C3c and iC3b. **a**–**c** SPR experiments were performed with CR3 αI immobilized to the surface of a streptavidin (SA) Biacore sensor chip (Cytiva) through a 29-Å-long NHS-PEG4-Biotin spacer arm. **a** Sensorgram obtained with iC3b as the analyte (5 injections at 0.1–10.0 μ*M*). **b** Sensorgram obtained with C3c as the analyte (5 injections at 0.7–60.0 μ*M*). **c** Maximum RU value reached during each injection plotted vs. analyte concentration. The experimental data point is shown as a filled circle and the fitted line is shown in the same color. **d**–**e** SPR experiments were performed with iC3b immobilized to a CM5 sensor chip (Cytiva) via maleimide chemistry. **d** Sensorgrams obtained with the αI variants as analyte (5 injections at 0.32–26.0 μ*M*). **e** Maximum RU value reached during each injection plotted vs. analyte concentration; the experimental data point is shown as a filled circle and the fitted line is shown in the same color (top). Plot of the *K*_D_^app^ vs. variant (error bars represent the standard error of the mean, *N* = 3) (bottom). WT: wild-type. 1A: R261A/R293A. 2A: R216/K231A. 1A2A: R261A/R293A/R216A/K231A. iC3b: for comparison, the *K*_D_^app^ from **a** and **c** is also shown. All SPR experiments were conducted at least twice independently with similar results. Source data are provided as a Source Data file.

The observation that the CR3 αI domain is tightly bound to either the MG6–MG7 or the MG1–MG2 regions of iC3b prompted us to reconsider the interaction between C3c and the CR3 αI domain in terms of specificity and avidity. To this end, we immobilized CR3 αI on a streptavidin (SA) sensor chip (Cytiva) by functionalizing it with the EZ-Link *N*-hydroxysulfosuccinimide (NHS)-polyethylene glycol (PEG)-biotin ester. This biotin labeling reagent has a 29-Å-long spacer arm that separates the small (22.2 kDa) CR3 αI domain from the carbohydrate layer of the sensor chip, thus allowing analyte recognition and binding to occur (Supplementary Fig. 11). Initial single-concentration experiments convinced us that iC3b, C3dg, and C3c bind to this surface, although a high level of CR3 αI domain immobilization was required to provide sensitive detection. Since surface attachment occurs at random amine groups on the protein surface, the high density of αI required for sensitive binding detection may be caused by the existence of preferential binding orientations incompatible with unhindered binding. Binding isotherms could be measured for iC3b between 0.1–10.0 × 10^−6^ *M* (Fig. 7a) and C3c in the concentration range 0.7–60.0 × 10^−6^ *M* (Fig. 7b). As saturation was not reached under the experimental conditions, in order to achieve a sense of the relative affinities of iC3b and C3c for CR3 αI, we used the maximum RU values at the end of each injection in a steady-state affinity model to approximate the respective affinity values (*K*_D_^app^) for these interactions (Fig. 7c). In agreement with previous reports, the interaction between the CR3 αI domain and iC3b is stronger with a *K*_D_^app^ ~5.2 × 10^−6^ *M*. In contrast, the interaction between the CR3 αI domain and C3c is about one order of magnitude weaker (*K*_D_^app^ ~3.9 × 10^−5^ *M*). The maximum binding response achieved for iC3b is ~1.5-fold greater than that for C3c based on the fit-predicted RU_max_ values at saturation, which is close to the ratio of their molecular masses (~1.2, assuming 173.2 kDa for iC3b and 139.0 kDa for C3c). This proportionality shows that the binding sites on the CR3 αI domain are equally accessible for the two analytes regardless of their binding strength. In terms of our crystallographic structure for iC3b–CR3 αI, the weaker interaction with C3c results from the interaction through the MG ring only. In comparison, the tighter interaction with iC3b is explained by the simultaneous recruitment of two semi-independent interaction surfaces on the MG ring and the canonical interaction motif present in the TED domain.

To test the relevance of the two C3c binding sites identified on CR3 αI by X-ray crystallography, we expressed and purified two sets of αI variants with mutations on residues predicted to contribute to either interaction surface and determined their effect on the *K*_D_^app^ for iC3b by SPR (Fig. 7d–e). Both the reference CR3 αI and most of the assayed mutants were marginally stable when probed by circular dichroism (CD) thermal denaturation, with melting temperatures in the range 41–47 °C (Supplementary Fig. 12). Mutations targeting the iC3b^U^–CR3 αI interaction surface caused, on average, less disruption to the αI fold stability than mutations targeting the iC3b^D^–CR3 αI interaction surface. Indeed, several mutants targeting the latter interaction surface were so severely destabilized that they could not be produced in soluble form or concentrated for binding assays. For the affinity ranking of the mutant proteins, we immobilized iC3b on the surface of a CM5 sensor chip through Cys988^iC3b^ in a quasi-physiological state with thiol coupling chemistry^10^. The sensor chip was first tested with CR3 αI as the analyte, yielding the same *K*_D_^app^ (7.1 × 10^−6^ *M*). However, the mutant proteins showed a consistent decrease in binding affinity, which was slightly greater for mutations targeting the MG1–MG2 surface (2A, R216A/K231A) than for mutations involving the MG6–MG7 surface (1A, R261A/R293A) (Fig. 7e). A variant that combined both sets of mutations experienced a greater binding impairment than either parental protein (1A2A, R216A/K231A/R261A/R293A). Taken together, these results indicate that the interaction surface characterized in the iC3b^U^–CR3 αI complex might be the dominant interface on C3c in vivo. The fact that SPR mimics some of the physiological properties of an opsonized surface (e.g., surface attachment of iC3b, locally high concentration of the opsonin) supports our proposal. However, other factors (e.g., interactions mediated by the rest of the CR3 ectodomain, tight receptor-ligand clustering, and lateral interaction of immobilized components on two approaching surfaces) might also play important roles in vivo.

Altogether, the interactions observed in the crystal structure of iC3b–CR3 αI are consistent in identity and strength with the affinity binding constants measured in solution by SPR. In the light of the crystallographic evidence, we can rationalize present and past SPR results. Albeit with a lower affinity, C3b can also bind the CR3 αI domain because the MG6–MG7 interaction surface and, to a lesser extent, the MG1–MG2 surface are partially solvent-exposed, therefore, accessible for binding. This is particularly so when iC3b, C3b, or C3c are immobilized on the sensor surface at a high concentration. In contrast, C3d shows a high-affinity interaction mode consistent with its known properties as an anchor for the CR3 αI domain^10^. Remarkably, surface-immobilized iC3b exhibited heterogeneous binding properties that can now be understood as the competition for the αI domain of two partially independent binding modules. When the αI domain is immobilized at high density, it is possible for the MG ring and the TED domains of two independent iC3b molecules to latch onto individual CR3 αI domain molecules, an avidity-enhancing event that cannot be easily reproduced on surfaces where the opsonin, rather than the receptor, is immobilized. The complementary views afforded by immobilization of either the opsonins, C3c, or the CR3 αI domain are relevant since, in vivo, the two molecules are bound to their respective cell surfaces.

### Structural basis for iC3b positive selectivity for CR3

Conversion of C3b to iC3b involves a profound structural transformation that underpins the functional switch from complement activation to becoming a ligand for integrin receptors (CR3/CR4). The drive for the negative selectivity of C3b toward CR3 has already been explained by the presence of a well-folded CUB domain in C3b that precludes binding of CR3 by steric hindrance^10^ (Supplementary Fig. 13). The crystal structure of iC3b–CR3 αI provides insights that reveal the drivers for the positive selectivity of iC3b toward CR3.

**Fig. 8:**
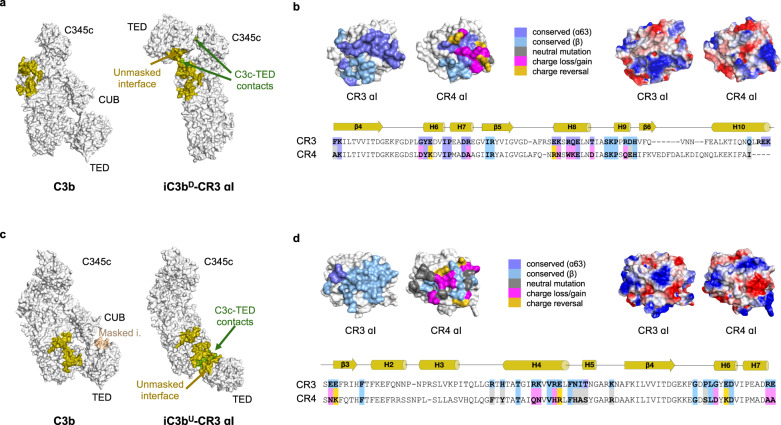
Structural basis for iC3b selectivity toward integrin receptors. **a** Molecular surface representations of C3b (PDB 2I07) and iC3b^D^ roughly in the same orientation. CR3 αI has been removed from the complex with iC3b^D^ to reveal the binding site. CR3 αI binding site is mapped onto the molecular surface of iC3b^D^ in olive color. The same residues have been highlighted in C3b for comparison. Additionally, on iC3b^D^, the contact area between the C3c moiety and the TED in its final position is shown in green. iC3b^D^ residues which were inaccessible to CR3 αI in C3b but have become exposed in iC3b^D^ are denoted as unmasked interface. **b** Like **a**, comparison of C3b (PDB 2I07) and iC3b^U^. Residues on C3b that could not bind CR3 αI due to steric impediments or lack of rotational freedom are mapped in wheat color and denoted as masked i(nterface). Upon conversion to iC3b^U^, residues in the masked interface on C3b are mapped onto iC3b^U^ as part of the unmasked interface. **c** Sequence variation and electrostatic properties of the αI domains of CR3 and CR4 at the contact interface with the C3c moiety of iC3b, shown as molecular surfaces in the same orientation. The view is perpendicular to the iC3b^D^–CR3 αI interface. Top left, sequence variation mapping. Residues that are not part of the interface are shown in white. Interfacial residues strictly conserved in CR4 αI are colored according to which chain of iC3b^D^ they interact with: light blue (chain β); slate (chain α63). Residues not conserved are classified (and color-coded) according to: nonconservative substitutions without a change in net charge (gray); substitutions with loss or gain of net charge (magenta); charge reversal substitutions in either direction (gold). Top right, electrostatic potential mapped on the molecular surface, calculated with APBS^44^. Bottom, sequence alignment of mapped regions with identical color code. **d** Like **c**, view is perpendicular to the iC3b^U^–CR3 αI interface.

Structural analysis shows that the two critical factors driving the positive selection of iC3b for CR3 are the exposure of novel interaction surfaces in iC3b and the cooperation between the C3c moiety and the TED domain. In C3b, part of the CR3 αI binding motif of the TED domain is hidden and sterically blocked by the CUB domain, thereby explaining the absence of a C3b–CR3 interaction (Fig. 8a,b). Likewise, surfaces employed by the TED domain to interact with the C3c moiety of iC3b^U^ or iC3b^D^ are also inaccessible in C3b because of steric or geometric impediments derived from the relatively stiff connection with the CUB domain (Fig. 8a,b). This, combined with the fact that the C3c binding sites for CR3 αI are low-affinity sites, explains the requirement for an unlocked TED domain that can cooperate with the C3c moiety to create composite binding surfaces for CR3 αI. Additionally, for the iC3b^U^–CR3 αI complex to assemble, the MG1 surface near the tight turn between β-strands C–D (residues 41−44), which is masked in C3b, must become exposed in iC3b to enable the binding of CR3 αI to the MG1–MG2 domains (Fig. 8b).

To act as a bimodular docking platform for CR3, the steric and geometric coupling between the C3c moiety and the TED domain in iC3b must be released by loosening the tether that connects them. Another critical requirement for functional independence is that they do not reassociate with higher affinity than the individual complexes with CR3 αI. Accordingly, in iC3b^D^–CR3 αI, the surface area buried at the TED–C3c moiety interface is 59 Å^2^ vs. 466 Å^2^ buried inside the TED–CR3 αI interface. Similarly, in iC3b^U^–CR3 αI, the larger TED–C3c moiety interface (382 Å^2^) is still significantly smaller than the interfaces with CR3 αI: 874 Å^2^ (C3c moiety) and 466 Å^2^ (TED domain).

Inspection of the iC3b–CR3 αI interaction surfaces rules out the possibility that either the CUB remnants or the CUB^f1^ segment of C3dg may present a steric impediment for CR3 αI (or full CR3) binding. Firstly, the TED–CR3 αI interface orientates the C-terminal end of the TED domain so that the CUB remnants, including the CUB^f1^ segment, can leave the complexes without interfering with CR3 binding. This is true of both iC3b^D^–CR3, where the TED domain is positioned by the C345c domain, and iC3b^U^–CR3, where the TED domain moves through a considerably shorter path (~27 Å). Secondly, analysis of the iC3b^U^–CR3 αI and iC3b^D^–CR3 αI complexes in this light shows that the rearrangement from C3b to iC3b has not only removed steric impediments on the TED domain but has also accomplished inter-domain rearrangements compatible with binding the entire CR3 headpiece and consistent with the function of the iC3b–CR3 complex in immune phagocytosis. Finally, molecular dynamics of models of the two iC3b–CR3 αI complexes containing the complete CUB^g^ segment confirm that the ensembles are stable and compatible with the presence of relaxed conformations of the long CUB^g^ tether (Supplementary Methods and Supplementary Fig. 14).

### Specific residue substitutions explain differential recognition of iC3b by CR3/CR4 αI

CR3 and CR4 are homologous β_2_ integrin receptors that share iC3b as their primary ligand on opsonized surfaces. Negative stain electron microscopy studies have proposed a binding site for CR4 αI between the MG3–MG4 domains of iC3b^20^. Remarkably, this proposed binding site for CR4 αI does not overlap with either of the two binding sites for CR3 αI identified in this work despite considerable sequence similarity between their respective αI domains (47.6%/73.0% sequence identity/similarity)^20^.

Superposition of the CR4 αI domain crystal structure (PDB 1N3Y)^28^ onto the iC3b^D^–CR3 αI and the iC3b^U^–CR3 αI complexes sheds light on the structural and sequence bases for the differential recognition of iC3b by CR3/CR4. As expected, the αI structures can be accurately superimposed (RMSD 1.4 Å over 176 Cα atoms). Analysis of the interface residues in both complexes revealed multiple substitutions in CR3 residues that make polar/charged contacts with iC3b (Fig. 8c,d). The number and electrostatic nature of the amino acid substitutions are more striking for iC3b^U^–CR3 αI (Fig. 8d) but also significant for iC3b^D^–CR3 αI (Fig. 8c). Thus, in iC3b^D^–CR3 αI, four out of seven (>50%) residues contributing polar/charged interactions to the interface with iC3b are exchanged: E253^CR3^ > K251^CR4^, K279^CR3^ > N277^CR4^, Q282^CR3^ > K280^CR4^ and R293^CR3^ > Q291^CR4^ (Fig. 8c). Two of these substitutions (K279^CR3^ > N277^CR4^ and Q282^CR3^ > K280^CR4^) could be considered conservative reciprocal exchanges. All substitutions are scattered over the three α-helices involved in the interaction of the αI domain and are predicted to reduce affinity.

For the iC3b^U^–CR3 αI complex, the amino acid differences between CR4 and CR3 αI domains appear to cause greater disruption to the interface. They are predicted to abolish binding: seven out of 12 residues (~60%) have been replaced, many by charge reversal or charge removal mutations (Fig. 8d). The exchanged residues are: E179^CR3^ > K177^CR4^, K217^CR3^ > N215^CR4^, R220^CR3^ > H218^CR4^, N224^CR3^ > H222^CR4^, E258^CR3^ > M256^CR4^, R261^CR3^ > A259^CR4^ and E262^CR3^ > A260^CR4^. These mutations spread over the MG1–MG2 interacting face of CR3 αI and involve helices α4, α6, and α7 (the most C-terminal of which is in contact with α-helices involved in iC3b^D^ recognition) and the only solvent-exposed edge of the central β-sheet of the αI domain (Fig. 8d).

## Discussion

The crystal structure of iC3b in complex with the αI domain of CR3 that we present here sheds light on some critical yet unresolved issues regarding how leukocyte-specific integrin receptors recognize complement-opsonized surfaces. The crystallographic asymmetric unit can be interpreted into three distinct iC3b–CR3 αI interaction surfaces. The first complex contains an extended conformation for the CUB^g^ linker, stabilized by a few crystalline contacts, connecting the MG7 domain of the C3c moiety with the TED domain at the end of the tether (Fig. 1d). Our structure of iC3b describes a representative conformation for the CUB^g^ segment and is in agreement with previous proposals based on biochemical and functional assays^17^, NS-EM analysis, and with the SAXS data presented here^18,19^. Furthermore, it substantiates the notion that iC3b is composed of two semi-independent modules, the C3c moiety and the TED domain, which cooperate to perform functions that are hard to accomplish in an uncoordinated fashion. Tellingly, the specific recognition of iC3b by CR3, CR4, and even CR2^29^, have in all cases implicated residues from both the TED domain and the C3c moiety.

In addition to the extended complex conformation, the crystal structure reveals two interaction surfaces between the αI domain of CR3 and the C3c moiety of a crystallographically neighboring iC3b molecule (iC3b^U^–CR3 αI and iC3b^D^–CR3 αI), which had not been previously identified. One of the interfaces encompasses the MG6–MG7 domains and defines an upside-down conformation for iC3b (iC3b^D^–CR3 αI), while the other engages the MG1–MG2 domains and defines an upright conformation (iC3b^U^–CR3 αI). The name (upright vs. upside-down) refers to the orientation that the C3c subunit of iC3b adopts with respect to the target surface relative to the most probable pose for C3b. Based on homology modeling of the CR3 headpiece, both complexes seem to mediate effective recognition of opsonized surfaces by interacting with the complete CR3. Using alternative low-affinity docking sites for CR3 αI on the MG ring of iC3b, besides the high-affinity TED binding site, should be beneficial for iC3b by raising the overall affinity for CR3 (as shown by SPR) as well as by increasing the robustness of the interaction.

Recognition of iC3b on opsonized surfaces by CR3, therefore, appears to be a complex process involving potentially several orientations of the C3c moiety and TED domains of iC3b and distinct recognition surfaces on the C3c moiety, MG1–MG2 in iC3b^U^ and MG6–MG7 in iC3b^D^. Recognition by CR4 of the iC3b opsonin adds another layer of complexity because available evidence indicates that CR4 αI recognizes structural features present in MG3–MG4^20^. Therefore, CR3 and CR4 together nearly saturate all possible binding sites consisting of contiguous pairs of MG domains. Furthermore, our analysis of the interactions between the MG6–MG7 and MG1–MG2 domain pairs and CR3 αI, and modeling of CR4 αI at the same position, indicate that specific residues critical for iC3b recognition on the surface of CR3 αI have been substituted in CR4 αI to the effect that interaction with the MG6–MG7 and MG1–MG2 binding sites would be reduced or eliminated. Taken together, the structure of the iC3b–CR3 αI complex provides important information to understand the positive selectivity of iC3b for CR3 and, at the same time, helps to explain how CR4 αI succeeds in avoiding competition for CR3 αI binding sites.

The coexistence in solution and crystalline form of several conformations of free and complexed iC3b allows us to finally explain and reconcile previous electron microscopy- and SAXS-based observations made for the structure of iC3b, on the one hand, and the low-resolution shapes calculated for iC3b–CR3 complexes, on the other hand. The extended-conformation iC3b found in the asymmetric unit indicates that the complex exists in a flexible state where the C3c moiety and the TED domain can move semi-independently (Fig. 1d). In contrast, the iC3b^U^–CR3 αI complex presents a markedly different architecture where the TED domain is found near the MG ring’s base, which results in a more elongated shape for the complex (Fig. 1e–f). Finally, the structure of iC3b^D^–CR3 αI has a compact organization with the TED domain placed close to the MG7 and C345c domains (Fig. 1g–h). This continuum of conformational states, which are accessible to iC3b and the iC3b–CR3 αI complex, explains the variety of observations that have been made about the structures of these species at low concentrations by electron microscopy and SAXS^18–20^.

For now, it remains challenging to conclusively discriminate which one of the two complexes, iC3b^U^–CR3 αI and iC3b^D^–CR3 αI, might be more relevant in vivo since both of them appear to fulfill many theoretical and experimental requirements for functional relevance. In particular, both iC3b conformations can be used to build clash-free models of the complex of iC3b with the upper part of the CR3 headpiece, encompassing the αI domain and the β-propeller domain of the α chain and the βI and hybrid domains of the β chain, either directly (without manual adjustments) when iC3b^U^–CR3 αI is used as the base structure, or, with iC3b^D^–CR3 αI, after modest rotations around the very flexible connection between the α chain αI and β-propeller domains (Fig. 5 and Supplementary Fig. 7). Furthermore, experimental validation by SPR of the two interaction surfaces suggested by the crystal structure of iC3b–CR3 αI shows that disrupting either interface reduced binding affinity, even though the disruption to binding affinity seems to be greater for mutations targeting the iC3b^U^–CR3 αI interaction surface (Fig. 7). Further evidence supporting the physiological role of the iC3b^U^–CR3 αI comes from previous NS-EM data of the interaction between iC3b and the CR3 headpiece^20^. In these studies, the authors located a secondary interaction site between the C345c knob of iC3b and the β-propeller/βI domain of the CR3 headpiece, which would match our homology model for the iC3b^U^–CR3 headpiece interaction.

Additionally, the C3c moiety of the two iC3b–CR3 αI complexes remains either strictly (iC3b^D^) or approximately (iC3b^U^) aligned with the direction normal to a hypothetical opsonized surface. In this orientation, the C3c moiety could help to orientate the iC3b–CR3 complex along the same normal direction by laterally interacting with both the TED domain, the CR3 αI domain, and additional domains of the CR3 headpiece to strengthen the interaction and increase the number of ligand-integrin receptor complexes per unit area. We hypothesize that facilitating the alignment of iC3b–CR3 complexes may be the most crucial function accomplished by decoupling the C3c moiety from the TED domain, which provides the tightest-interaction interface for CR3 αI. In this role, the C3c subunit would act as a standing and leveling aid, a function that cannot be easily performed by the smaller and more spherical TED domain alone.

Further support in favor of the iC3b^U^–CR3 αI interface comes from the observation that an *N*-linked glycosylation appears to be necessary for CR3 binding since bovine serum conglutinin can inhibit it^4^. Bovine serum conglutinin is a collectin-type lectin whose structure has been recently elucidated^30^. Conglutinin has a strong preference for *N*-acetylglucosamine (GlcNAc) but can also bind to some complex-type and high-mannose-type oligosaccharide chains. The interaction of conglutinin and iC3b can be efficiently inhibited by GlcNAc^31^. Fittingly, the iC3b α-oligosaccharide is of high-mannose type^32,33^, and conglutinin binding is proposed to occur through the terminal α (1–2)-mannobiosyl unit^34^. There are three *N*-linked glycosylated residues in C3 and iC3b, Asn63, Asn917, and Asn1595^35–39^. Of them, only Asn63^iC3b^ is found at one of the C3c-αI interfaces that have been identified, corresponding with iC3b^U^–CR3 αI. Given its proximity to the αI domain (Supplementary Fig. 8), it is conceivable that conglutinin binding to Asn63^iC3b^ could disrupt the interaction between iC3b and CR3, as observed experimentally. These observations, in combination with the fact that the side chain of Asn224^CR3^, the only glycosylated residue in the αI domain of CR3, points directly toward the binding interface, lend support to the proposal that iC3b^U^–CR3 αI may represent the likelier conformation in vivo.

**Fig. 9:**
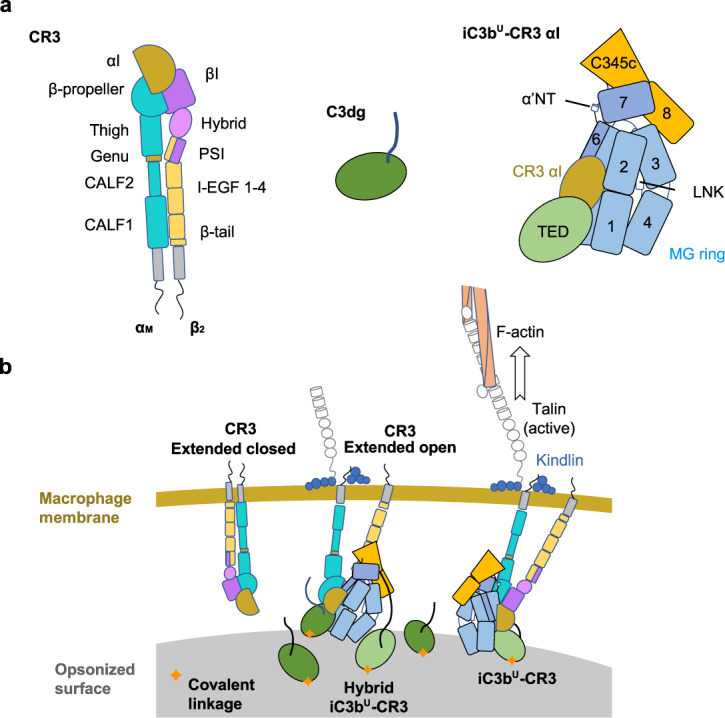
Structural integrative model of the iC3b–CR3 interaction. **a** Schematic representation of CR3 (extended closed conformation), C3dg, and iC3b (upright conformation). Domains and salient features are labeled. **b** Model of the iC3b–CR3 interaction across the gap between a macrophage (brown band, top) and a heterogeneous iC3b- and C3dg-opsonized surface (grey surface, bottom). iC3b and C3dg are covalently attached to the opsonized surface via a covalent bond involving the Gln991 carbonyl ester (orange star). Two types of iC3b–CR3 (extended open conformation) complexes are depicted: a hybrid complex whereby the TED domain and the C3c subunit in contact with the same CR3 come from different but adjacent iC3b molecules; and a binary heterocomplex involving a single iC3b molecule. The short intracellular region of CR3 is shown interacting with talin and kindlin in some complexes, which in turn recruit the force-producing actomyosin cytoskeleton responsible for the pulling forces necessary for phagocytosis.

We hypothesize that an outstanding feature of the two compact iC3b–CR3 αI complexes characterized in this work is that they do not need to be assembled from modules (C3c moiety and TED domain) belonging to the same iC3b molecule. Most lines of evidence based on diluted protein samples in solution or on electron microscopy grids indicated that the extended conformation would be the most probable conformation for the iC3b–CR3 αI and iC3b–CR3 complexes. However, the geometry of the two complexes is also compatible with the C3c moiety and the TED domain being donated by two neighboring iC3b molecules (as seen in the crowded environments of the crystal and the surface of the SPR sensor chips), or even with a scenario whereby the C3c moiety comes from one iC3b molecule and the TED domain from a C3dg molecule, thus forming a hybrid complex (Fig. 9). Colocalization within a certain distance threshold (<10 nm, based on the length of the CUB remnants and the two modes of interaction identified in this work) on a densely iC3b-opsonized surface would be sufficient to trigger this domain swapping, thereby resulting in a crisscrossing maze of C3c moiety-TED domain connections on the plane of the opsonized surface. Since integrins typically form clusters comprising 900–5400 molecules/µm^2^ ^40^, clustered CR3 could interact with a similarly packed layer of iC3b molecules. Under these crowded conditions, all iC3b molecules in the cluster would be tightly packed with average intermolecular distances below 10 nm, a distance within reach of the CUB remnants. Furthermore, opsonized surfaces are extremely heterogeneous systems with a complex composition that can change dynamically. Unless prevented, the continuous factor I-catalyzed conversion of iC3b to C3dg on opsonized surfaces will result in mixed populations of opsonins with increasing ratios of C3dg over iC3b. These mixed populations might feature hybrid iC3b–C3dg–CR3 complexes, as suggested above. It is interesting to consider that CR3 may have evolved the capacity to recognize iC3b and C3dg either independently or as part of hybrid complexes to ensure the proper identification of opsonized particles under heterogeneous conditions.

In the widely accepted model of mechanosensitive integrin activation (or outside-in activation), only ~1% of cell-surface-exposed unliganded integrins adopt the extended-closed or extended-open conformations, which allow ligand binding with affinities typically thousands of times greater than the closed conformations^41^. For the iC3b–CR3 interaction to correctly mediate adhesion of blood flow-exposed leukocytes to iC3b-opsonized particles, the ligand (iC3b) must bind the integrin receptor (CR3) while the intracellular tail of the β_2_ subunit of CR3 is engaging the force-generating actomyosin cytoskeleton via talin/kindlin^41^. Therefore, successful adherence, phagocytosis, and other effector functions depend on the rapid stabilization of iC3b–CR3 complexes. C3dg is known to mediate CR3-dependent phagocytosis by macrophages but only at much higher concentrations than iC3b, therefore suggesting that the C3c moiety is responsible for the enhanced recognition by CR3 and the ensuing phagocytosis^42^. The structural data presented on the iC3b^D^–CR3 αI and iC3b^U^–CR3 αI interaction surfaces provide a basis to understand the critical role of the C3c moiety of iC3b in stabilizing and strengthening the interaction with CR3 in the context of densely clustered receptor-ligand interactions beyond the contributions of the better-known interaction with the TED domain (or C3dg) (Fig. 9).

In conclusion, we have solved the crystal structure of the iC3b–CR3 αI and found that it includes the well-supported extended conformation previously shown by biochemical and low-resolution structural methods and two novel docking platforms for the TED–CR3 αI subcomplex on the C3c moiety. In combination with biophysical and binding data, these structural data shed light on previously unresolved issues: (i) the overall structure of iC3b and the position/orientation of the TED domain; (ii) the contribution of the C3c moiety to the recognition of iC3b by CR3; and (iii) the structural basis for the selectivity of CR3 for iC3b. Based on available data, we propose an integrative model that describes the structural basis of the dynamic processes by which iC3b-opsonized surfaces are recognized by CR3-harboring immune cells. This model should lead to a comprehensive understanding of immune adherence signaling processes where iC3b and CR3 are key actors.

## Methods

### Purification of iC3b

iC3b was produced from C3 purified from plasma as previously described^19^. Human C3 was purified from plasma containing 20 m*M* EDTA and 10 m*M* benzamidine. Supernatant of a 10% (w/v) sodium sulfate precipitation, dialyzed against 50 m*M* phosphate buffer (pH 7.0), 5 m*M* benzamidine, 10 m*M* EDTA, and 150 m*M* NaCl, was applied to a lysine-Sepharose column to remove plasminogen. The flow-through was collected, dialyzed against 20 m*M* Tris-HCl (pH 8.6), and loaded onto a HiPrep DEAE FF 16/10 anion exchange column (Cytiva). C3 was fractionated using a 0–350 m*M* NaCl gradient, and C3-containing fractions were pooled together and dialyzed against 20 m*M* sodium phosphate (pH 6.0), 40 m*M* NaCl, and applied to a Mono S HR 5/5 cation exchange column (Cytiva). Protein was eluted with a 0–500 m*M* NaCl gradient, and C3-containing fractions were pooled together and immediately processed to C3b by incubation with factor B and D in 10 m*M* HEPES-NaOH (pH 7.5), 150 m*M* NaCl, and 2 m*M* MgCl_2_ for 30 min at 37 °C, and then further processed to iC3b by addition of factor H and factor I in the same buffer for 30 min at 37 °C. Finally, iC3b was re-purified on a MonoQ HR 5/5 anion exchange column (Cytiva) and polished by gel filtration on a Superdex200 10/300 GL column (Cytiva) previously equilibrated in 10 m*M* HEPES-NaOH (pH 7.5), 150 m*M* NaCl. Factors B, D, H, and I were purified from plasma by immunoaffinity^19^.

### Expression and purification of CR3 αI domain

A codon-optimized gene encoding residues 126–321 of the CR3 receptor inserted (αI)-domain of the α_M_ subunit (also von Willebrand A domain, VWA) with two stabilizing mutations (C128S/I316G) was ordered from GenScript and subcloned into the plasmid pETM-11 for expression of the recombinant protein in *Escherichia coli* BL21(DE3) as a tobacco etch virus (TEV)-cleavable N-terminal hexahistidine fusion. Expression was induced with 1 m*M* IPTG at 20 °C, and cells were harvested by centrifugation after overnight incubation. CR3 αI was purified from the soluble extract by Ni^2+^ affinity chromatography. The elution peak was dialyzed against imidazole-free buffer, and the hexahistidine tag was cleaved off by the addition of TEV (1:50 mass ratio). Untagged CR3 αI was further purified by subtractive Ni^2+^ affinity chromatography followed by size-exclusion chromatography over a Superdex 75 10/300 GL column (Cytiva) in 20 m*M* HEPES (pH 7.5), 200 m*M* NaCl. The following mutations in the protein-coding sequence were introduced by site-directed mutagenesis: K245A, E253A, K245A/K279A, K245A/E253A, R216A/K231A, R261A/R293A, R216A/K217A/R220A/K231A, and K245A/R261A/H295A/R293A. All mutant proteins were expressed, and those variants that could be obtained in soluble form were purified and concentrated as previously described. The folding and thermal stability of the reference sequence and the mutant variants were assessed by circular dichroism (CD) in a Jasco J-715 spectropolarimeter at a single temperature (25 °C) and by thermal denaturation at 222 nm between 25 and 95 °C (temperature ramp speed of 1 °/min, data acquisition rate of one data point every 0.5°).

### Purification of iC3b–CR3 αI complex

The iC3b–CR3 αI complex was formed by mixing purified iC3b with CR3 αI at a 1:2 molar ratio and chromatographed over a Superdex 200 10/300 GL column (Cytiva) in 20 m*M* HEPES-NaOH (pH 7.5), 200 m*M* NaCl, 5 m*M* MgCl_2_ to separate the complex from excess unbound CR3 αI.

### Crystallization

The iC3b–CR3 αI complex was crystallized at 8 mg/mL by sitting-drop vapor-diffusion in 48-well plates by mixing 1 μL protein with 1 μL of the crystallization condition [100 m*M* Tris-HCl (pH 8.0), 8% (w/v) polyethylene glycol (PEG) 8000]. Crystals grew as long, thin needles, which were cryoprotected with 15% (v/v) sterile glycerol before being snap-frozen in liquid nitrogen.

### Data collection and processing

Numerous flash-frozen crystals were diffracted at 100 *K* at the BL13-XALOC (ALBA synchrotron source, Barcelona, Spain)^43^ and the microfocus PX2A (Soleil French national synchrotron facility, Paris, France)^44^ beamlines. Two data sets to 3.4-Å resolution were used for structure determination, but only the highest-quality data set, collected at the microfocus beamline, is described here. Complete diffraction data were measured on PX2A at 100 *K* with fixed wavelength (*λ* = 0.9801 Å) on an EIGER X 9 M area detector, indexed, and processed with *XDS*^45^ or *DIALS*^46^, and scaled and merged with Aimless^47^ from the *CCP4* software suite^48^. The data set belonged to the monoclinic space group *P*2_1_ with unit-cell dimensions *a* = 111.6 Å, *b* = 151.1 Å, *c* = 111.7 Å (α = γ = 90°, β = 92.8°). In keeping with current standard practice^49–51^, we chose to set the highest-resolution limit of the data set to 3.4 Å by including all reflection intensities in resolution shells with CC(½) > 15% (Supplementary Table 1).

### Structure determination

The crystal structure of iC3b–CR3 αI was determined by the molecular replacement (MR) method using the program *PHASER*^52^ from the *PHENIX* program suite^53^. The following search models were used: C3c (PDB 2A74)^54^, the TED domain from a C3b structure (PDB 2I07)^15^, and CR3 αI from the structure of the TED–CR3 αI (PDB 4M76)^10^. The TED and CR3 αI models were used as independent ensembles, not as a preformed TED–CR3 complex despite being available (PDB 4M76)^10^. The initial placement of the model was improved by rigid-body refinement with phenix.refine^55^.

### Model building and crystallographic refinement

After molecular replacement, cycles of maximum likelihood refinement with *REFMAC5*^56^ and validation-guided manual building in *Coot*^57^ were iterated to build a complete model of the iC3b–CR3 αI complex. Five % of randomly selected reflections were set aside for cross-validation. The final model contains 99.9% of residues in favored and allowed zones of the Ramachandran plot.

### Small-angle X-ray diffraction

Synchrotron SAXS was performed at the BM29 BioSAXS beamline at the European Synchrotron Radiation Facility (Grenoble, France)^58,59^ in continuous-flow batch mode at 4 °C. Further details are presented in the Supplementary Methods.

### Biosensor affinity/Binding affinity assays using SPR

Surface plasmon resonance (SPR) interactions experiments were performed on a Biacore X100 instrument (Cytiva). Depending on which protein was immobilized on the sensor chip, two experimental setups were used. To immobilize CR3 αI, it was first biotinylated with the EZ-Link *N*-hydroxysulfosuccinimide (NHS)-polyethylene glycol 4 (PEG4)-biotin ester (ThermoFisher Scientific ref. 21330) at a 1:2.5 molar ratio of protein to NHS-PEG4-biotin. Next, biotinylated CR3 αI was captured on a streptavidin (SA) sensor chip as instructed by the manufacturer at an immobilization level of ~900 RU. Analytes (C3c or iC3b) were flowed across the CR3 αI surface at five different concentrations between 0.1–10.0 × 10^−6^ *M* (iC3b) or 0.7–60.0 × 10^−6^ *M* (C3c). To screen the αI mutant variants, we immobilized iC3b on CM5 sensor chips using maleimide coupling chemistry to favor a pseudo-physiological anchoring via the reactive Cys988 of iC3b. In all cases, complex formation was examined in 10 m*M* HEPES-NaOH (pH 7.4), 100 m*M* NaCl, and 5 m*M* MgCl_2_. Finally, the surface was regenerated with 50 m*M* Na_2_EDTA and 1 *M* NaCl. Data were collected at 25 °C at a flow rate of 10 μL/min and were double referenced (data from a reference cell and a buffer injection were subtracted) to control for bulk refractive index changes. Data were evaluated using Biaevaluation X100 evaluation software (v. 1.1) (Cytiva), and affinity binding isotherms were analyzed by steady-state analysis.

### Molecular dynamics

All molecular dynamics (MD) simulations were carried out with GROMACS *v*. 2020.3^60^. Molecular systems consisting of iC3b^U^–CR3 αI or iC3b^D^–CR3 αI plus the CUB^g^ motif (residues 913–954) and the intact connections with the MG7 domain or the TED domain (in total, residues 907–971) were created by manual editing of coordinate files with *Coot*^57^, and the stereochemistry of the edited model was verified with MolProbity^61^. Further details are presented in the Supplementary Methods.

### Molecular surfaces

Molecular surfaces were calculated using the software PyMOL (https://pymol.org). Electrostatic potential mapping on molecular surfaces was calculated with APBS (Adaptive Poisson-Boltzmann Solver)^62^.

### Reporting summary

Further information on research design is available in the Nature Research Reporting Summary linked to this article.

## Supplementary information

Supplementary Information

Reporting Summary

## Data Availability

Atomic coordinates and crystallographic structure factors were deposited in the Protein Data Bank (PDB) under accession codes PDB 7AKK. Source data are provided with this paper.

## Source data

Source Data
