# Supplementary material for: The crystal structure of iC3b-CR3 αI reveals a modular recognition of the main opsonin iC3b by the CR3 integrin receptor
Source: Nat Commun. 2022 Apr 12;13:1955. doi: 10.1038/s41467-022-29580-2 (PMC9005620; doi:10.1038/s41467-022-29580-2)
Supplement: Supplementary file 1 — Supplementary Information [file 41467_2022_29580_MOESM1_ESM.pdf]

Supplementary Information for

The crystal structure of iC3b-CR3 αI reveals a modular recognition of the main opsonin iC3b by the CR3 integrin receptor

Francisco J. Fernández*[1,2], Jorge Santos-López[1], Rubén Martínez-Barricarte[1,3,#], Javier Querol-García[1,2], Héctor Martín-Merinero[1,3], Sergio Navas-Yuste[1], Martin Savko[4], William E. Shepard[4], Santiago Rodríguez de Córdoba[1,3], M. Cristina Vega[1]*

[1] Centro de Investigaciones Biológicas Margarita Salas, Agencia Estatal Consejo Superior de Investigaciones Científicas, 28040 Madrid, Spain; [2] Abvance Biotech srl, 28003 Madrid, Spain; [3] Centro de Investigación Biomédica en Enfermedades Raras, 28040 Madrid, Spain; [4] Synchrotron SOLEIL, L'Orme des Merisiers Saint-Aubin, 91192 Gif-sur-Yvette, France.

[#] Present address: Division of Genetic Medicine, Department of Medicine, Vanderbilt University Medical Center, Nashville, TN 37232, United States.

* Corresponding authors: Francisco J. Fernández, M. Cristina Vega.

Email: cvega@cib.csic.es, fjfernandez@abvance.com

ORCIDs: 0000-0003-0628-8378 (MCV), 0000-0002-5015-1849 (FJF).

Classification

Biological sciences/Structural biology/X-ray crystallography

Biological sciences/Immunology/Complement cascade

Keywords

Complement, Opsonin proteins, iC3b, Integrin, CR3

Supplementary Methods

Small-angle X-ray diffraction. Synchrotron SAXS measurements ($I(s)$ *vs.* s, where $s = 4\pi \sin\theta/\lambda$, 2θ is the scattering angle, and $\lambda = 0.992$ Å) were performed at the BM29 BioSAXS beamline at the European Synchrotron Radiation Facility (Grenoble, France)[1,2] in continuous-flow batch mode at 4 ºC. Supplementary Table 2 summarizes the SAXS data and provides additional information. SAXS intensity data were recorded from 30 μL samples at several different concentrations across the ranges 1.4−5.5 mg/mL (iC3b-CR3 αI complex) and 0.17−0.7 mg/mL (CR3 αI domain) and from a matching solvent blank. The individual 2D-data frames were radially averaged to produce unsubtracted 1D $I(s)$ *vs.* s scattering profiles. For the final data reduction process statistical checks were performed to discard frames affected by radiation damage or systematic scaling errors. Data were averaged, buffer subtracted, and merged to produce the final SAXS profile for each species. The *ATSAS* 2.8 software package[3] and BioXTAS *RAW*[4] were used to extract structural information and perform *ab initio* shape restoration. Firstly, the number of Shannon channels and maximum usable s was estimated with *SHANUM*[5]. The extrapolated forwarding scattering at zero concentration $I(0)$ and the radius of gyration R_g were evaluated using the Guinier approximation ($\ln I(s)$ *vs.* s^2) and from the real-space pair-distance distribution function ($P(r)$ *vs.* r) calculated with *GNOM*[6]. From the $P(r)$ profile the maximum particle dimension (D_{max}) could be evaluated. Two separate concentration-independent methods were used to estimate the molecular mass of the iC3b-CR3 αI complex and the free αI domain: the volume of correlation (V_C)[7] and the empirical correction to the Porod volume (V_p)[8] using the *ATSAS* dattools *DATMOW*, *DATVC*, and *DATPOROD*. *DATCLASS* classified the SAXS derived shapes as compact and potentially unique[9]. *Ab initio* shape restoration was performed using dummy beads from 50 independent runs of *DAMMIF*[10], which were averaged with *DAMAVER*, clustered with *DAMCLUST*[8], and further refined with *DAMMIN*[11] to create the final *ab initio* shape. In each case, 20–40 individual dummy bead (*DAMMIF*) models were selected that fitted the data as judged by the reduced χ^2 test and the correlation map (CorMap) p-value; values of $\chi^2 = 1$ and $p > 0.01$ indicate the absence of any systematic discrepancy between the data and the fitted model[12].

Individual models were aligned with *SUPCOMB* with a threshold on the normalized spatial discrepancy (NSD) of 0.5, i.e. NSD < 1 for similar aligned models[13]. The final average model of the iC3b-CR3 αI domain have been produced at an estimated resolution of ~42 ± 3 Å with *SASRES*[14].

Molecular dynamics. All molecular dynamics (MD) simulations were carried out with GROMACS *v.* 2020.3[15]. Molecular systems consisting in $iC3b^{U}$-CR3 αI or $iC3b^{D}$-CR3 αI plus the CUB^{g} motif (residues 913–954) and the intact connections with the MG7 domain or the TED domain (in total, residues 907–971) were created by manual editing of coordinate files with *Coot*[16] and the stereochemistry of the edited model was verified with MolProbity[17]. For the protein, ligands (glycan moieties), and ions (solvent counterions to neutralize the system's net charge), the OPLS/AA force field[18,19] was used, and TIP4P[20] was used for the water.

All systems were first subjected to minimization, followed by 1-ns equilibration of the NVT and NPT ensembles. All production runs (1 μs each) were carried out in the NPT ensemble at 300 *K* and 1 bar. Temperature was controlled by Nosé−Hoover[21,22] (coupling constant τ_t = 0.1 ps) and pressure by Parrinello−Rahman[23,24] (τ_p = 2.0 ps) schemes. To avoid harsh density oscillations, the first 5 ns of the NPT equilibration run were performed with Berendsen weak coupling[25] for temperature (τ_t = 0.1 ps) and pressure (τ_p = 2.0 ps). Periodic boundary conditions were applied in three-dimensional space, and electrostatic forces were calculated with the Particle Mesh Ewald (PME) method[26,27] using a real-space cutoff of 1.2 nm and an FFT grid density of 6.25 nm^{-1}. Lennard-Jones interactions were truncated at 1.2 nm. Covalent bond lengths in the protein and ligands were constrained to their reference values with P-LINCS[28]. SETTLE was used to constrain the water geometry[29]. Equations of motion were integrated using the leapfrog scheme with a time step of 2 fs. During the first 1 ns of this run, all protein heavy atoms were harmonically restrained to their initial positions. The latter trajectories were analyzed, and the structures were clustered using the GROMOS algorithm[30]. Multiple related clusters were detected for $iC3b^{U}$-CR3 αI or $iC3b^{D}$-CR3 αI, whose central structure allowed the calculation of root-mean-square fluctuations (RMSF, in Å) per residue during the trajectory (Supplementary Fig. 10a,c). A surface

representation of each representative structure confirms that relatively modest displacement of the CUB^g around its equilibrium position in either complex (Supplementary Fig. 10b,d). Although the segment does not adopt stable secondary structures, we refer to the CUB^g in these models as “stable” in the sense that it retains its overall shape and characteristics for the duration of the MD runs. During the simulation, we detected the formation of a few contact points between the CUB^g-containing segment and other iC3b or CR3 αI surfaces, which appear to provide some additional stabilization energy to the complex.

Data availability. Source data are provided with this paper.

Supplementary Figures and Tables

Supplementary Table 1. Data collection and refinement statistics (molecular replacement).

	iC3b-CR3 αI
**Data collection**	
Space group	$P2_1$
Cell dimensions	
a, *b*, *c* (Å)	111.29, 150.73, 111.30
α, β, γ (°)	90, 92.80, 90
Resolution (Å)	53.77−3.39 (3.52−3.39) *
$I / \sigma I$	4.03 (0.53)
R_{merge}	0.338 (2.422)
$CC_{1/2}$	0.973 (0.167)
Completeness (%)	99.62 (96.71)
Redundancy	3.6 (3.6)
**Refinement**	
Resolution (Å)	53.77−3.39 (3.52−3.39)
No. reflections	50 383 (4556)
R_{work} / R_{free}	0.1921 / 0.2294
No. atoms	24 012
Protein	23 866
Ligand/ion	125
Water	21
B-factors	
Protein	112.46
Ligand/ion	124.06
Water	56.80
R.m.s. deviations	
Bond lengths (Å)	0.013
Bond angles (º)	1.60

The structure was determined from a single crystal. * Values in parenthesis are for the highest-resolution shell.

Supplementary Table 2. Interactions observed between the TED domain of iC3b and CR3 αI. Interactions and other properties of the interface(s) were calculated with *PISA*[31]. The interface area is 465.6 Å^2, the solvation energy is −1.2 kcal/mol, the total binding energy is −5.6 kcal/mol, and the hydrophobic p-value is 0.5. The number of hydrogen bonds is 10, and there are no salt bridges or disulfide bridges. Distances are shown in Å.

**iC3b-CR3 αI**		**iC3b**	**Dist. (Å)**	**CR3 αI**
**Type of interaction**	**#**	**C3dg (TED)**		**CR3 αI**
Hydrogen bonds	1	Lys1195 NZ	3.3	Leu205 O
	2	Lys1195 NZ	3.2	Leu206 O
	3	Arg1232 NH2	3.7	Ile145 O
	4	Arg1232 NH2	3.8	Gly143 O
	5	Asp1225 OD1	3.4	Gly143 N
	6	Asp1225 O	3.3	Ser144 N
	7	Asp1225 OD1	2.7	Ser144 N
	8	Asp1225 OD1	2.4	Ser144 OG
	9	Asp1225 OD2	3.8	Thr209 N
	10	Asp1225 OD2	2.8	Thr209 OG1
No Salt bridges				

Supplementary Table 3. Interactions observed between the C3c moiety of iC3b^{U} and CR3 αI or the TED domain. Interactions were calculated with *PISA*[31]. This interface is a composite interface made up of residues from the iC3b β chain with a minor contribution from the C3c α' fragment 1. Concerning the C3c moiety-CR3 αI domain interface, the interface area is 873.5 Å^2, the solvation energy is 2.4 kcal/mol, the total binding energy is −3.0 kcal/mol, and the hydrophobic p-value is 0.8. The number of hydrogen bonds is 8, the number of salt bridges is 5, and there are no disulfide bridges. Interactions are broken down for every individual chain in iC3b, C3 τ (23–667), C3c α' fragment 1 (749–954), C3dg (including the TED domain) (955–1303), and C3c α' fragment 2 (1321–1663). Distances are shown in Å.

**iC3b^{U}-CR3 αI**		**iC3b chain**	**Dist. (Å)**	**CR3 αI/TED**
**Type of interaction**	**#**	**C3 β**		**CR3 αI**
Hydrogen bonds	1	Thr67 OG1	2.3	Lys217 NZ
	2	Ser157 O	3.1	Arg261 NH2
	3	Asn162 O	2.5	Lys231 NZ
	4	Asn162 OD1	3.1	Lys235 NZ
	5	Gln161 NE2	3.6	Val219 O
	6	Gln161 NE2	2.7	Phe223 O
	7	Asn71 ND2	2.2	Pro249 O
	8	Ser159 OG	2.5	Glu262 OE2
Salt bridges	1	Glu15 OE1	3.7	Arg216 NE
	2	Glu15 OE1	3.2	Arg220 NH1
	3	Glu15 OE1	3.5	Arg216 NH1
	4	Glu15 OE2	3.7	Arg216 NH2
	5	Glu15 OE2	2.7	Arg216 NH1
		**C3c α' frg. 1**		
No Hydrogen bonds				
No Salt bridges				
		**C3c α' frg. 2**		
Not at the interface				
		**C3 β**		**TED**
Hydrogen bonds	1	Gln87 OE1	2.5	Asn1186 ND2
No salt bridges				

Supplementary Table 4. Interactions observed between the C3c moiety of iC3b^D and CR3 αI or the TED domain. Interactions were calculated with *PISA*[31]. This interface is a composite interface made up of residues from the iC3b β chain and residues from the iC3b α' fragment 1 chain. For the C3c moiety-CR3 αI domain interaction, the interface area is 836.4 Å^2, the solvation energy is −3.5 kcal/mol solvation energy, the total binding energy is −7.0 kcal/mol, the hydrophobic p-value is 0.4. There are 9 hydrogen bonds and 2 salt bridge but no disulfide bridges. Interactions are broken down for every individual chain in iC3b, C3 β (23–667), C3c α' fragment 1 (749–954), C3dg (including the TED domain) (955–1303), and C3c α' fragment 2 (1321–1663). Distances are shown in Å.

**iC3b^D-CR3 αI**		**iC3b**	**Dist. (Å)**	**CR3 αI/TED**
**Type of interaction**	**#**	**C3 β**		**CR3 αI**
Hydrogen bonds	1	Gln557 OE1	3.9	Arg293 NH1
	2	Gln558 N	3.0	Lys290 O
No Salt bridges				
		**C3c α' frg. 1**		
Hydrogen bonds	1	Asn738 N	3.2	Glu253 OE1
	2	Asn738 OD1	2.9	Lys245 NZ
	3	Ser743 O	4.0	Arg261 NH
	4	Glu737 O	3.5	Lys245 NZ
	5	Glu737 O	3.8	Tyr252 N
	6	Ile733 O	3.4	Lys279 NZ
	7	His897 O	2.5	Gln282 NE2
Salt bridges	1	Glu744 OE1	4.2	Arg261 NH2
	2	Lys891 NZ	3.7	Glu253 OE1
		**C3c α' frg. 2**		
Not at the interface				

Supplementary Table 5. Small-angle X-ray scattering (SAXS) statistics.

(*a*) Sample details			
	iC3b	CR3 αI	iC3b-CR3 αI
Organism	*Homo sapiens*	*Homo sapiens*	*Homo sapiens*
Source	iC3b was produced by activation of native C3	Synthetic gene, expressed recombinantly in *E. coli*	Reconstituted *in vitro* at a 1:2 molar ratio and purified by SEC
Description	Uniprot ID P01024 (CO3_HUMAN)	Uniprot ID P11215 (ITAM_HUMAN), residues 126−321 with two stabilizing mutations (C128S/I316G)	N/A
Theoretical extinction coefficient at 280 nm in water ε^{280} (M^{-1} cm^{-1}) [1]	171 200 (*ox*)<br>169 950 (*red*)	4470 (no Trp/Cys)	175 670 (*ox*)<br>174 420 (*red*)
Molecular mass *M* from composition (Da) [1,2]	173 247 (*p.s.*)<br>~184 000 (*glyco*)	22 189 (*p.s.*)	195 418 (*p.s.*)<br>~206 189 (*glyco*)
Concentration (range/values) measured	N/A	1.4−5.5 mg/mL (1.4, 2.75 and 5.5 mg/mL)	0.17−0.7 mg/mL (0.17, 0.35 and 0.7 mg/mL)
Solvent composition	N/A	20 m*M* HEPES (pH 7.5), 200 m*M* NaCl	20 m*M* HEPES (pH 7.5), 200 m*M* NaCl, 5 m*M* $MgCl_2$

(*b*) SAS data collection parameters	
Source, instrument and description or reference	BM29 BioSAXS beamline at the European Synchrotron Radiation Facility (Grenoble, France)[1,2]
Wavelength (Å)	0.992
Sample-to-detector distance (m)	2.849
q-measurement range ($Å^{-1}$)	0.003–0.50
Method for monitoring radiation damage	Visual inspection of data frames
Exposure time, number of exposures	1.0 s, 10 exposures in total
Sample configuration	30 μL
Sample temperature (ºC)	4.0

(*c*) Software employed for SAS data reduction, analysis and interpretation	
SAS data reduction	*ATSAS* v2.8[3]
Guinier, *P*(*r*), scattering particle volume	*ATSAS* v2.8[3] / BioXTAS RAW[4]
Shape/bead modeling	*DAMMIF/N*, *DAMCLUST* (*ATSAS* v2.8)
Atomic structure modeling (rigid)	*SASREF* (*ATSAS* v2.8)
Modeling of missing sequence from PDB files	N/A
Molecular graphics	PyMOL (https://pymol.org/)

(*d*) Structural parameters		
Guinier analysis	CR3 αI	iC3b-CR3 αI

$I(0)$ (cm^{-1})	20.7	192.5
R_g (Å)	23.8	55.0
q-range ($Å^{-1}$)	0.18−0.55	0.15−0.23
Quality-of-fit parameter (r^2 fit)	0.99	0.98
M (Da) from *I*(0) (ratio to expected value)	21 300 (0.96)	197 000 (1.00)
P(*r*) analysis		
$I(0)$ (cm^{-1})	19.8	192.0
R_g (Å)	21.7	56.8
D_{max} (Å)	60.0	190.0
q-range ($Å^{-1}$)	0.17−5.01	0.11−5.01
Quality-of-fit parameter (χ^2)	1.02	1.14
M (Da) from *I*(0) (ratio to expected value)	20 400 (0.92)	194 400 (0.99)
Volume (V_P / V_C)	0.5 / 0.3	1.7 / 1.3
(*e*) Shape modeling results		
	iC3b-CR3 αI	
q-range for fitting	0.11−1.39	
Symmetry/anisotropy assumptions	*P*1 / unknown	
χ^2 value/range	0.94−0.97	
Model resolution (Å)	42.0	
(*f*) Atomistic modeling		
	iC3b-CR3 αI	
Method	*SASREF* (*ATSAS* v2.8)	
q-range for fitting	0.11−5.01	
Symmetry/anisotropy assumptions	*P*1 / unknown	
Distance restraints	<189 Å between residues 910−971 iC3b	
χ^2 value/range	2.0−8.0	

[1] ProtParam, Expasy web server at https://web.expasy.org/cgi-bin/protparam/protparam. *ox*, all Cys oxidized to cystines. *red*, all Cys taken as reduced (free thiol form).

[2] Theoretical molecular mass calculated from the primary sequence (*p.s.*). Estimated molecular mass in the naturally occurring glycosylated form is indicated where necessary (*glyco*).

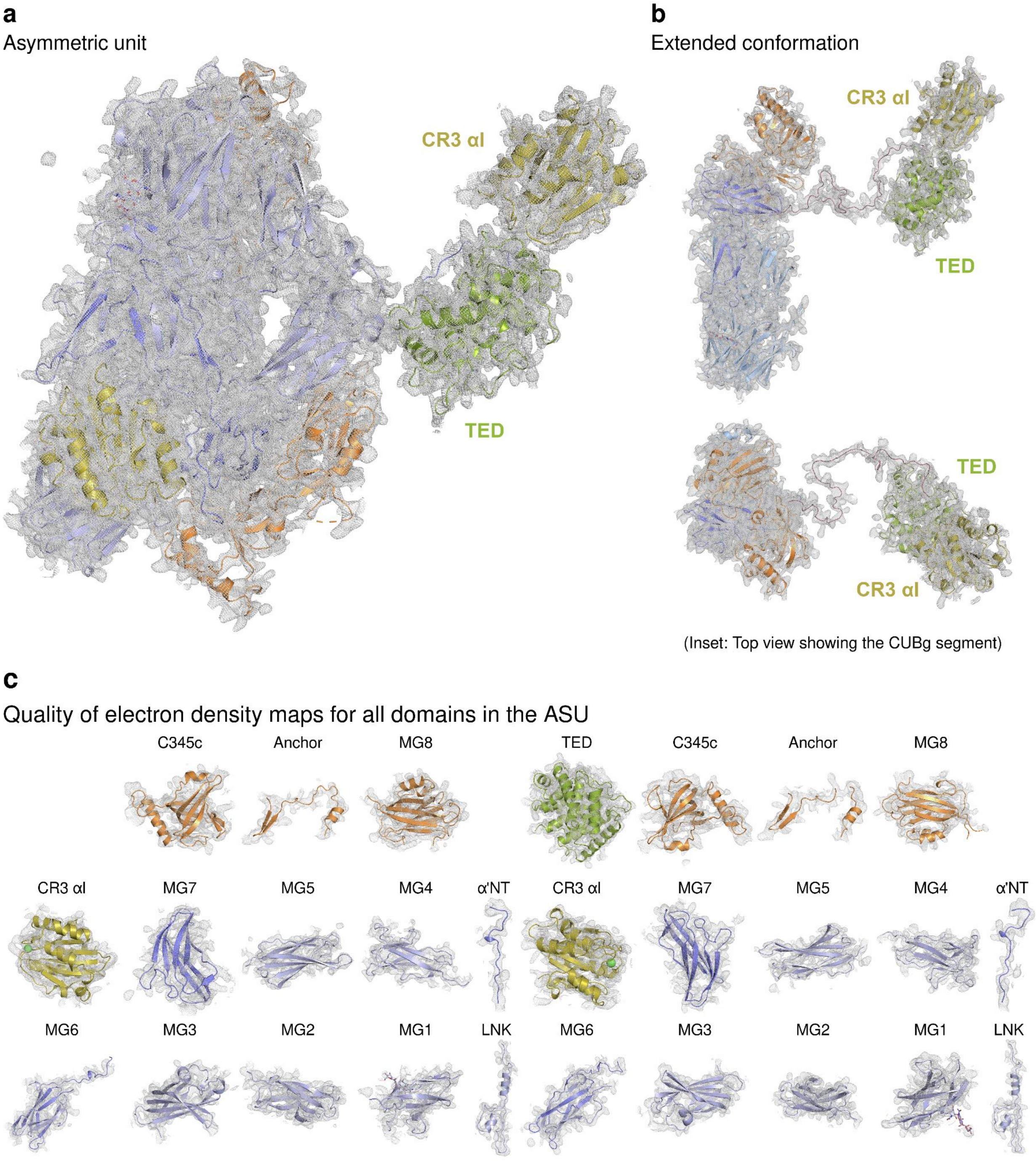

(Legend on the next page.)

Supplementary Fig. 1. Electron density maps of iC3b-CR3 αI, asymmetric unit, extended conformation, domains, and motifs. The overall quality of the electron density maps for the iC3b-CR3 αI crystal structure is shown for (**a**) the entire asymmetric unit, (**b**) the extended conformation of iC3b-CR3, and (**c**) all the domains and motifs present in the asymmetric unit. The remnants of the CUB domain connecting the MG7 and TED domains (CUB^g) could be traced for one iC3b subunit and are shown in (**b**), which is also shown in a top-view image on the inset. All maps are σ_A-weighted $2mF_O$-DF_C maps contoured at 1.0 σ level. The average map correlation coefficient (*MAPCC*) is 0.77–0.80 overall and it ranges from 0.67 (C345c in $iC3b^D$) to 0.84 (MG7 in $iC3b^D$ and MG2 in $iC3b^U$) across all domains. The *MAPCC* for the CR3 αI subunits is 0.75 and 0.79.

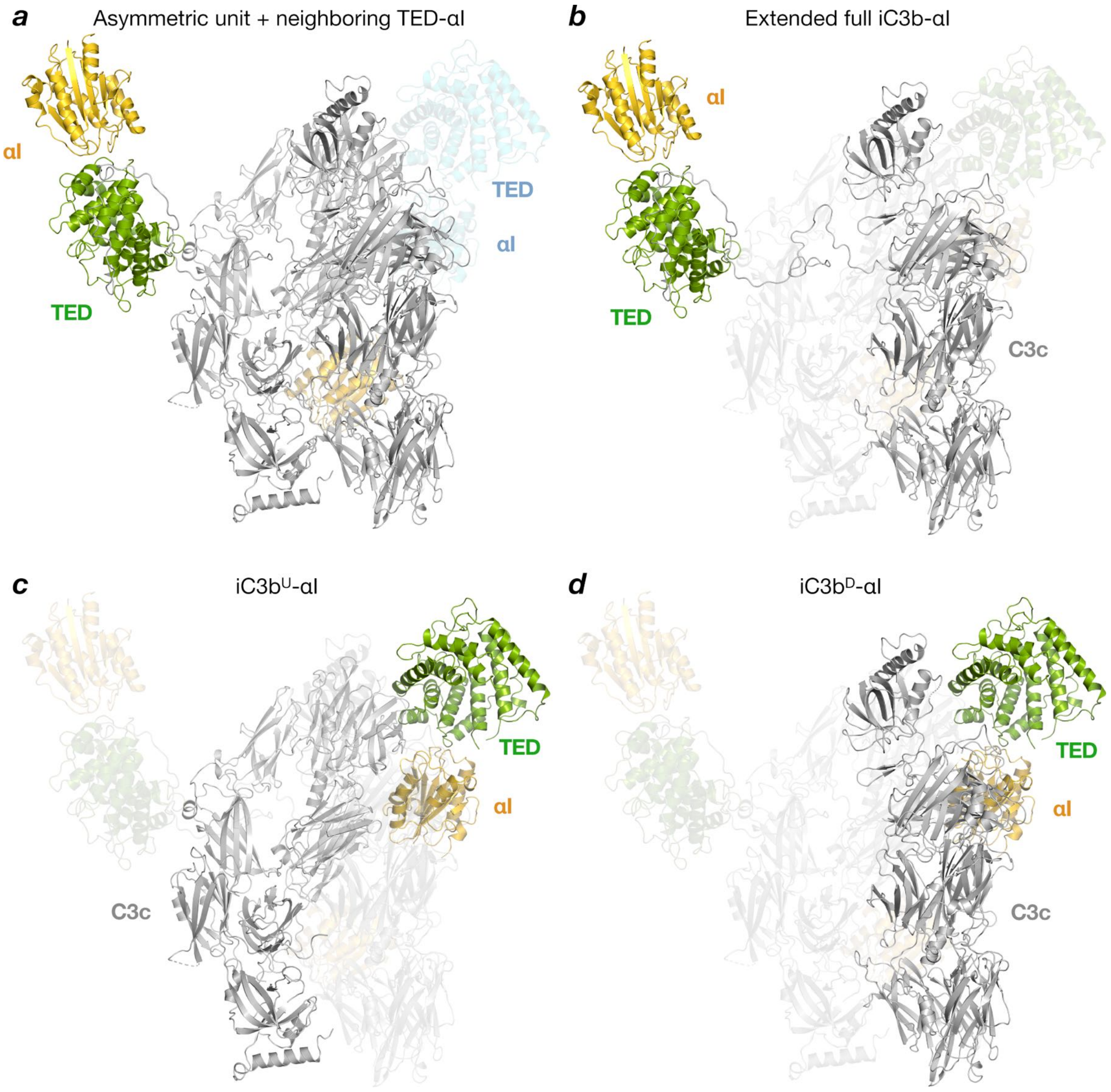

Supplementary Fig. 2. Different iC3b-CR3 αI complexes identified in the crystallographic structure. Cartoon representation of the contents of the asymmetric unit and selected subunits from crystallographic neighbors (in cyan). C3c is shown in grey, the TED domain in green and the CR3 αI domain in gold. (**a**) The asymmetric unit contains one copy of an iC3b TED-CR3 αI complex and a second copy of uncomplexed CR3 αI. A second TED-CR3 αI complex from a crystallographically equivalent molecule is shown in cyan. The same volume is shown in panels **b** to **d** with the same orientation as in **a.** (**b**) The extended form of the iC3b-αI complex is highlighted by making the component subunits opaque while the other subunits are transparent.

(**c**) iC3b^{U}-CR3 αI (upright conformation). (**d**) iC3b^{D}-CR3 αI (upside-down conformation).

Definitions of each conformation can be found in the main text.

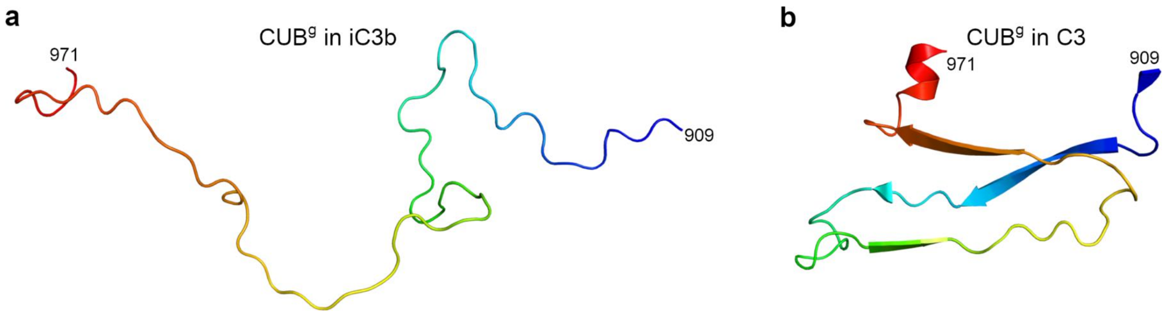

Supplementary Fig. 3. Unraveling of the CUB^g region (residues 909–971) of C3 in iC3b. Whereas the CUB^g portion of the CUB domain is fully folded in C3 (PDB 2A73 [http://doi.org/10.2210/pdb2A73/pdb])[32] (**b**), in iC3b the same region adopts an extended conformation as a result of the proteolytic release of the C3f fragment and the ensuing conformational destabilization of the CUB domain (**a**).

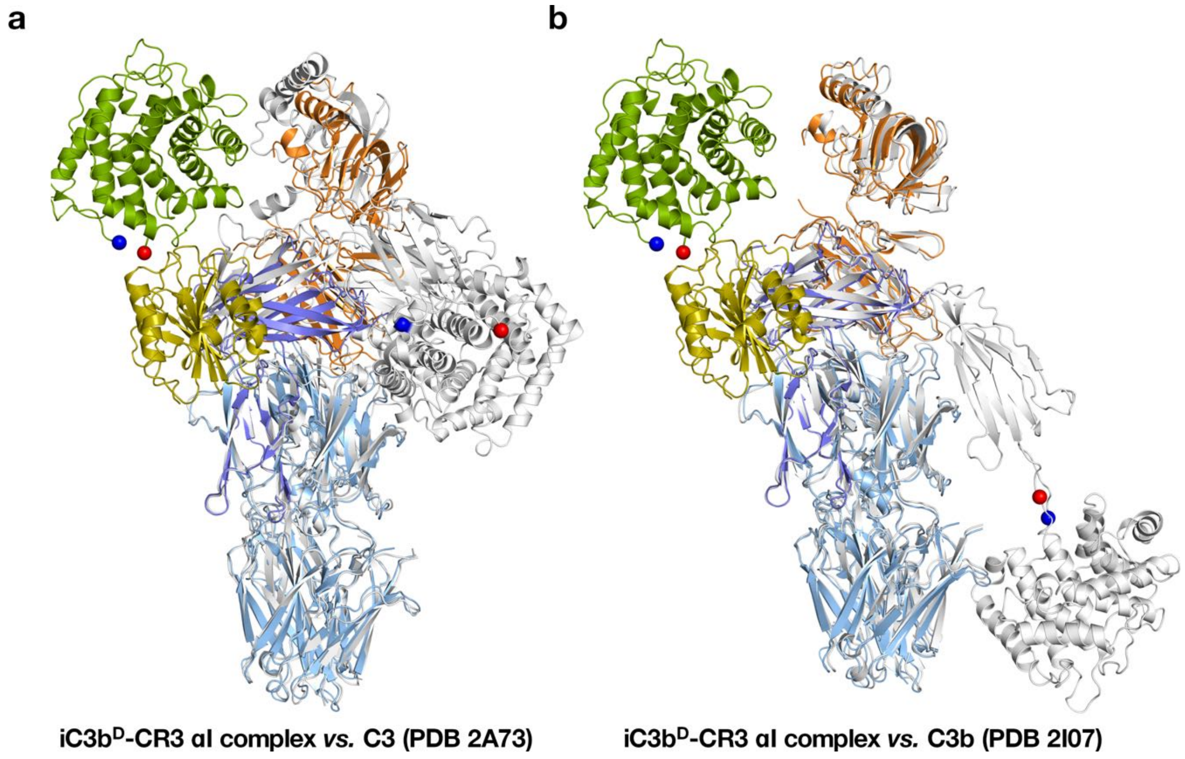

Supplementary Fig. 4. Superposition of the structure of iC3b^{D}-CR3 αI complex with those of C3 and C3b to show the relative position and orientation of the TED domain. The structure of iC3b^{D}-CR3 αI complex can be superposed with those of (**a**) C3 (PDB 2A73 [http://doi.org/10.2210/pdb2A73/pdb])[32] and (**b**) C3b (PDB 2I07 [http://doi.org/10.2210/pdb2I07/pdb])[33] with the secondary structure matching (SSM) algorithm as implemented in the CCP4 program *SUPERPOSE*[34] with root-mean-square displacements (RMSD) of 1.95 Å (722 Cα atoms) and 1.33 Å (1104 Cα atoms), respectively. The superposition algorithm did not consider non-superimposable regions as the TED domain and the CR3 αI subunit. The iC3b^{D}-CR3 αI complex is shown in the same color code used throughout the article, with the TED in green and the CR3 αI domain in olive. C3 and C3b are shown in white. The Cα atom of the N and C-terminal residues of the TED domain (residues 992–1287) are represented as blue and red spheres, respectively. Direct superposition of the TED domains of C3, C3b, and iC3b in the iC3b^{D}-CR3 αI complex structure with *LSQKAB*[35] shows that the transformation of the TED domain between C3b and iC3b in the iC3b^{D}-CR3 αI complex involves a translation of 74.7 Å

and a rotation around the centroid-connecting direction of 86.2º. Interestingly, the transformation necessary to move the TED domain from C3 to C3b is rather similar to the transformation required from C3 to iC3b (113.8 Å *vs* 109.2 Å, 95.1º *vs* 95.3º) but along two directions separated by 120º.

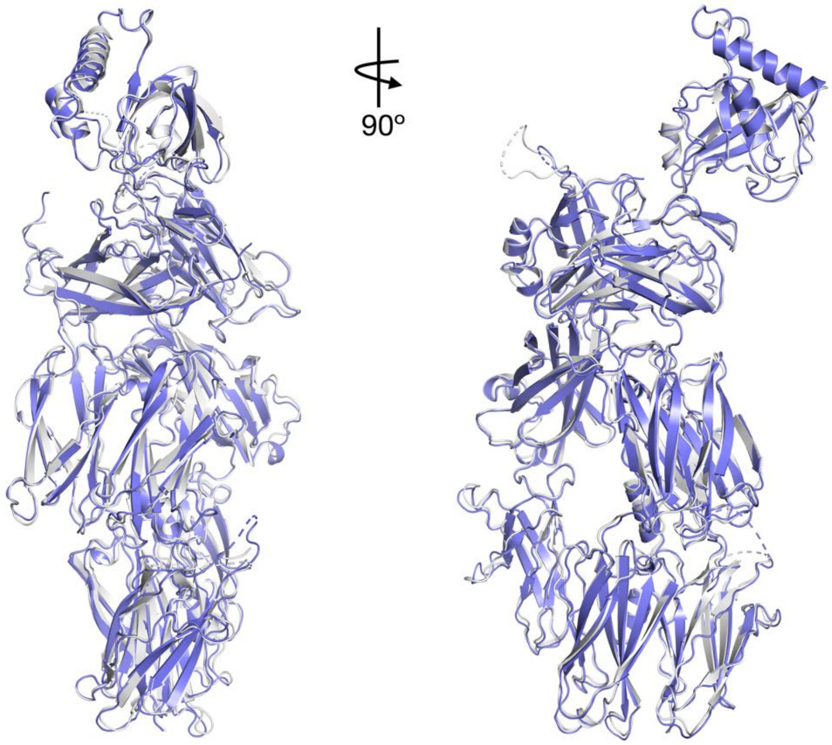

Supplementary Fig. 5. Similarity between the C3c moiety of the two iC3b-derived copies in the asymmetric unit. The two C3c moieties found in the asymmetric unit can be superimposed with the secondary structure matching (SSM) algorithm as implemented in the CCP4 program *SUPERPOSE*[34] with root-mean-square displacements (RMSD) of 0.76 Å (1113 residues). The superposition algorithm did not consider non-superimposable regions such as the TED domain and the CR3 αI subunits. The two views of the superposition are related by a 90º rotation around a vertical axis.

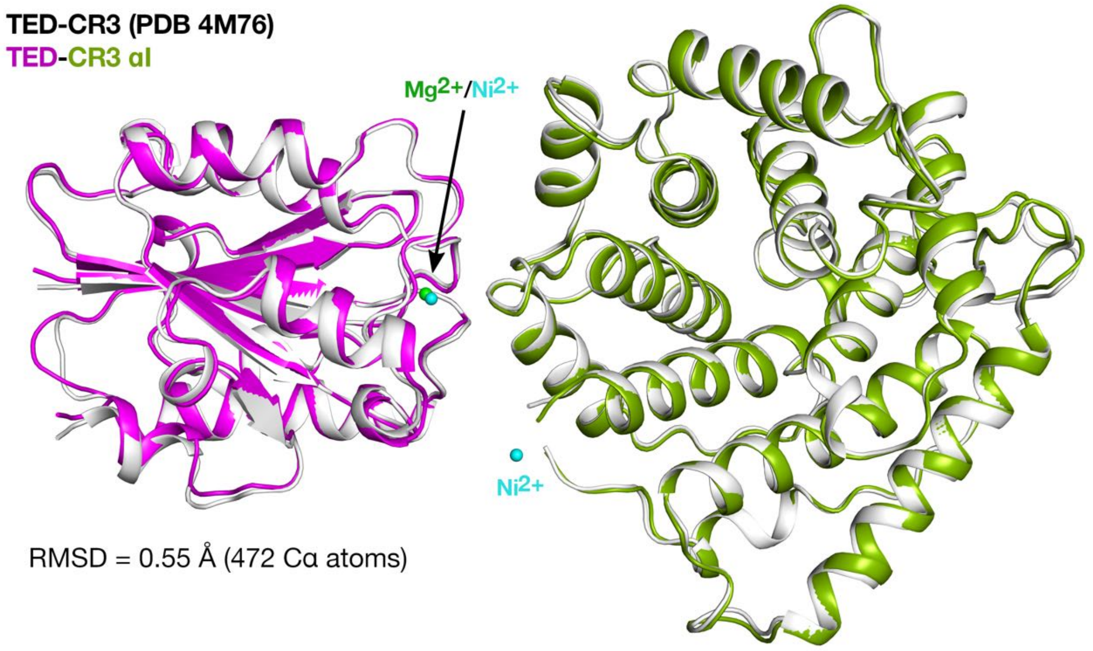

Supplementary Fig. 6. Comparison of the TED-CR3 αI domain interaction. Superposition of the TED-CR3 αI subcomplex extracted from the iC3b^{D}-CR3 αI complex (the TED domain is in green and CR3 αI in magenta) with the previously available TED-CR3 αI structure (both domains are shown in white) (PDB 4M76 [http://doi.org/10.2210/pdb4M76/pdb])[36]. The root-mean-square displacement (RMSD) between the two TED-CR3 αI complexes is 0.55 Å over 472 Cα atoms, indicating that there are no gross conformational changes of either the TED domain or the von Willebrand A domain of CR3 upon complex formation in the context of iC3b. While the structure of the C3d-CR3 αI complex contains Ni^{2+} in the MIDAS motif of CR3 αI, the structure of iC3b-CR3 αI complex contains the physiological cation, Mg^{2+}.

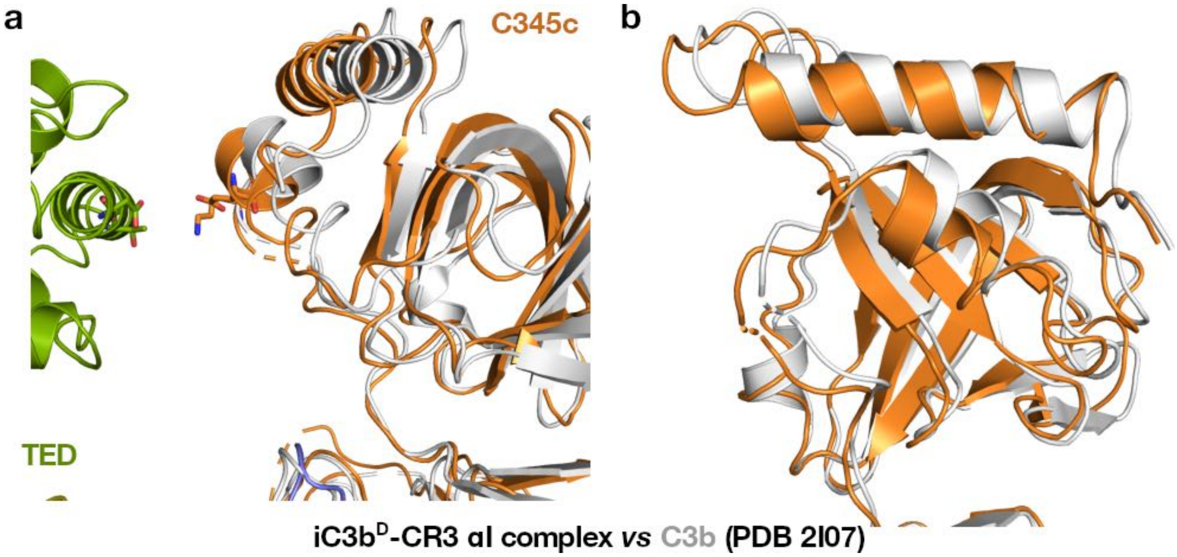

Supplementary Fig. 7. Close-up of the superposition of the iC3b^{D}-CR3 αI complex with C3b on the C345c domain (shown in Supplementary Fig. 4). a, Interface between the TED domain (in green) and the C345c (in orange) of the iC3b^{D}-CR3 αI complex with the corresponding region of C3b (in white)[33]. **b**, View of the superposition of the C345c domains of the iC3b^{D}-CR3 αI complex and C3b looking from the TED side (the TED has been removed for clarity). The most noticeable discrepancy between the iC3b^{D}-CR3 αI complexes and C3b barring the position of the TED domain concerns the C345c domain. As shown, the C345c in the iC3b^{D}-CR3 αI complex has rotated and laterally shifted its position towards the TED domain, with which it establishes a small but stable interaction. Even though the quantitative displacement of the C345c in the iC3b^{D}-CR3 αI complex is small (3 Å is the maximum linear displacement), and independent of crystal lattice contacts or the presence of TED. The latter observation argues in favor of an intrinsic structural change moving from C3b to iC3b, or an induced conformational change triggered by binding of CR3 αI.

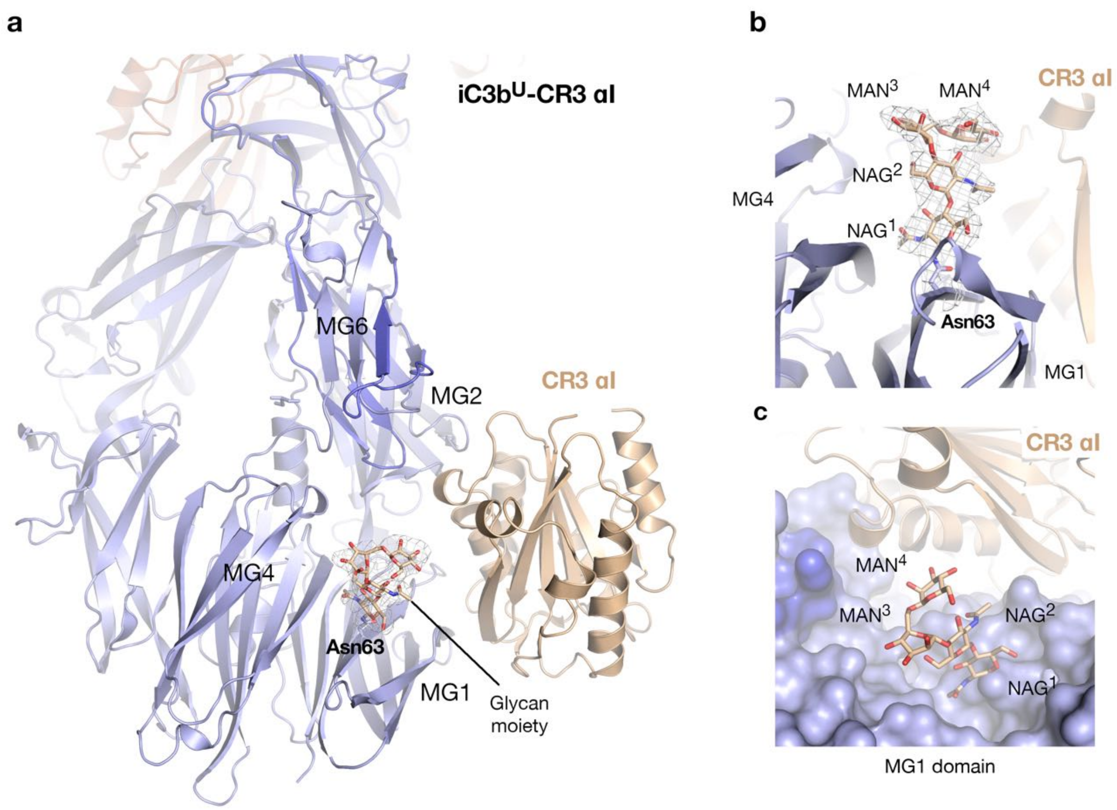

Supplementary Fig. 8. Glycosylation on Asn63 (iC3b^{U}). a, Position and orientation of the *N*-linked glycosylation on Asn63 within the MG ring of iC3b^{U}. Protein domains are shown in cartoons and color coded as in the main text. Note that the glycan moiety faces toward the interface with CR3 αI domain (in wheat). **b**, Close-up of the glycosylation. The electron density map for the glycan moiety is a σ_A-weighted $2mF_O–DF_C$ map depicted at 1.0 σ contour level. NAG, *N*-acetyl-glucopyranoside. MAN, mannopyranoside. MAN^3 is α-D-mannopyranoside, whereas MAN^4 is a β-D-mannopyranoside. Superscripted numbers refer to the sequential position of the glycan residue. **c**, MG1 represented as a molecular surface in chain color (light blue). The glycosylation sticks out from Asn63 into the interface cleft.

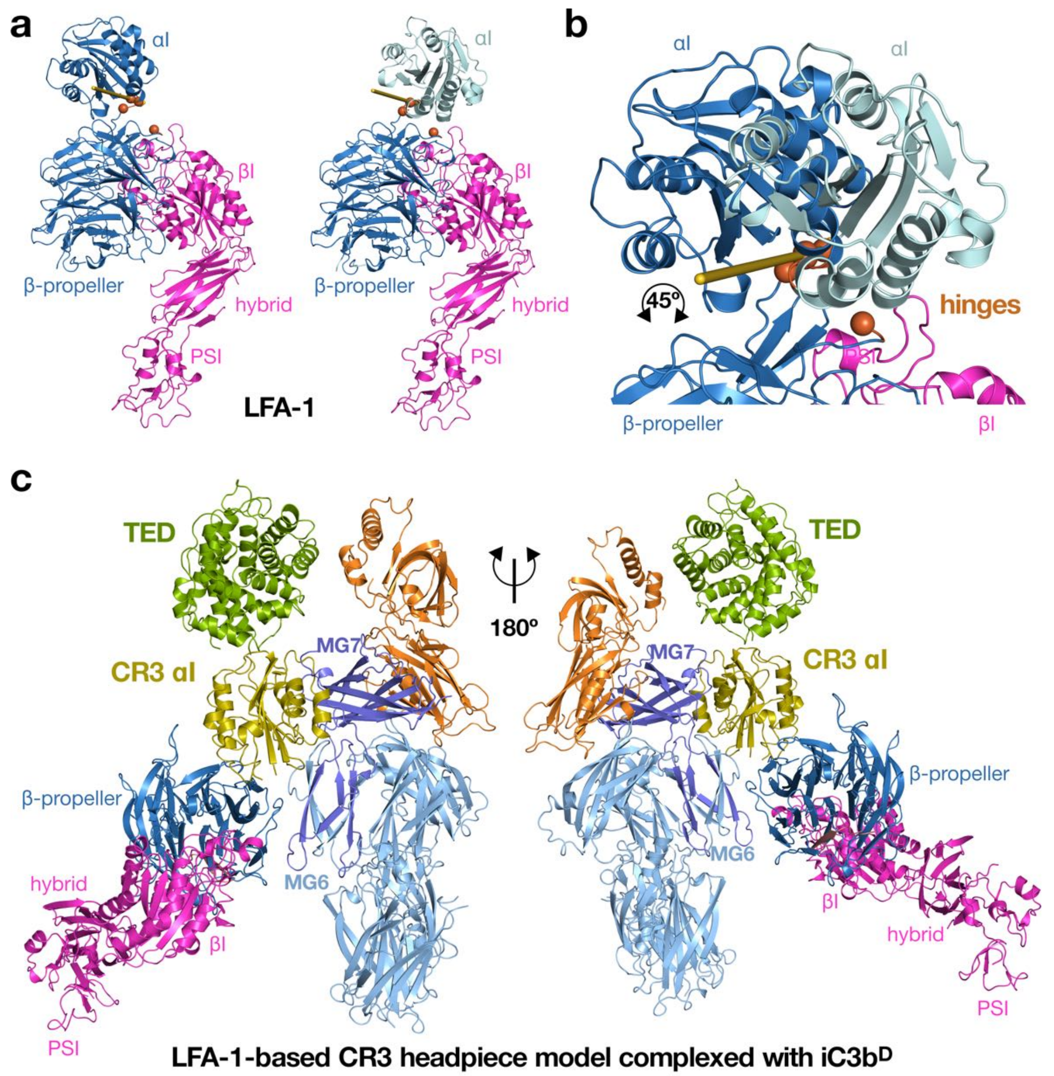

Supplementary Fig. 9. Compatibility of the iC3b^{D}-CR3 αI complex with the recognition by the CR3 headpiece. **a**, Domain motion analysis of the αI domain as observed in the structure of the LFA-1 headpiece (PDB 5E6S [http://doi.org/10.2210/pdb5E6S/pdb])[37], in blue, and after a 45º tilt introduced to accomplish a clash-free superposition of the LFA-1 headpiece (shown in sky blue and magenta) onto the iC3b^{D}-CR3 αI complex, in cyan. The domain motion analysis was performed with Dyndom[38]. The yellow arrow represents the rotation axis, and the orange segments the hinge regions. The ends of the hinge regions, which remained motionless, are shown as orange spheres. **b**, Detail of the 45º rotation relating the crystallographic orientation of the LFA-1 αI domain (blue) and the clash-free orientation (cyan). **c**, Suggested hybrid models

prepared by superposition of the CR3 αI subunit in iC3b^{D}-CR3 αI complex and the αI domain of LFA-1 (previously modeled as in panels **a** and **b**). Molecules are shown in cartoon representation and color coded as in the main text.

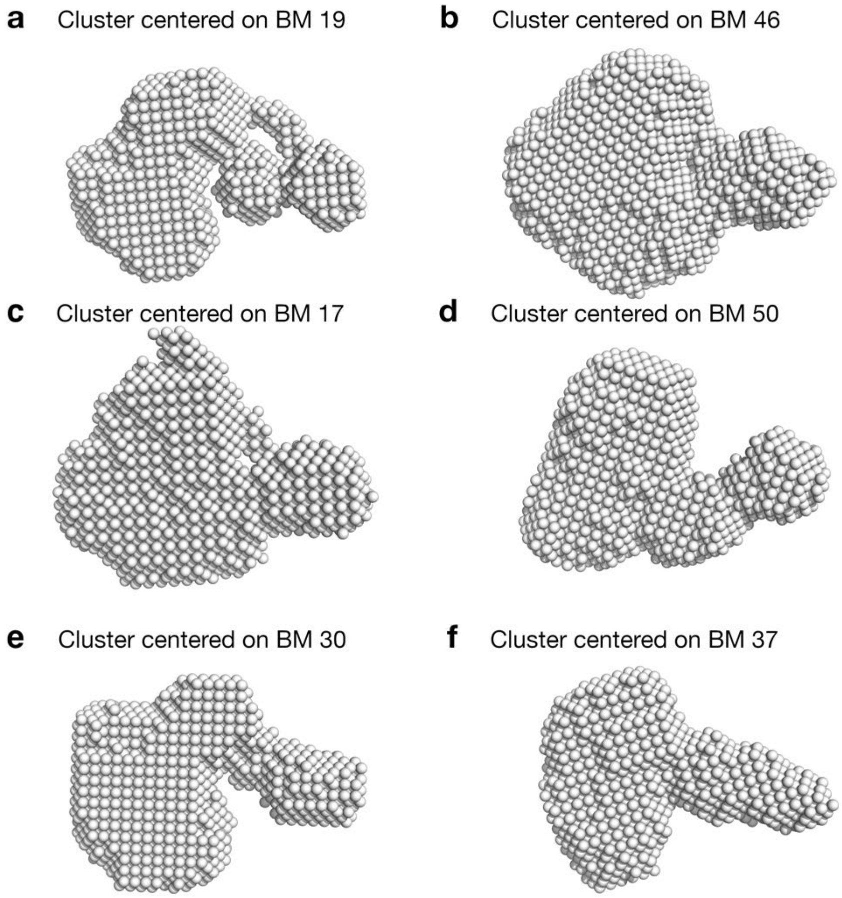

Supplementary Fig. 10. Representative bead models for the iC3b-CR3 αI complex in solution restored from SAXS scattering data. (**a**-**f**) Bead model (BM) representation of the center model for the six identified clusters of structurally similar models. BM were depicted with white spheres (radius 4.75 Å) and rendered with PyMOL (https://pymol.org). Clustering was performed on 50 bead models generated with *DAMMIF*[10] with *DAMCLUST*[8] using default parameters. Despite some variation, the salient features of the iC3b-CR3 αI complex (i.e., a large body connected to a slightly smaller, more elongated region) are conserved across all reconstructed shapes and clusters.

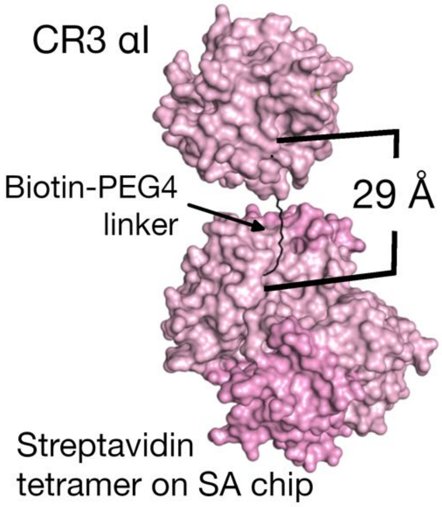

Supplementary Fig. 11. Design of the ligand for SPR experiments. The CR3 αI domain was immobilized on a streptavidin (SA) chip (Cytiva) after derivatization with an EZ-Link *N*-hydroxysulfosuccinimide (NHS)-polyethylene glycol (PEG4)-biotin ester. When outstretched, the linker has a maximum length of 29 Å thereby separating the ligand from the glycan layer on the sensor chip.

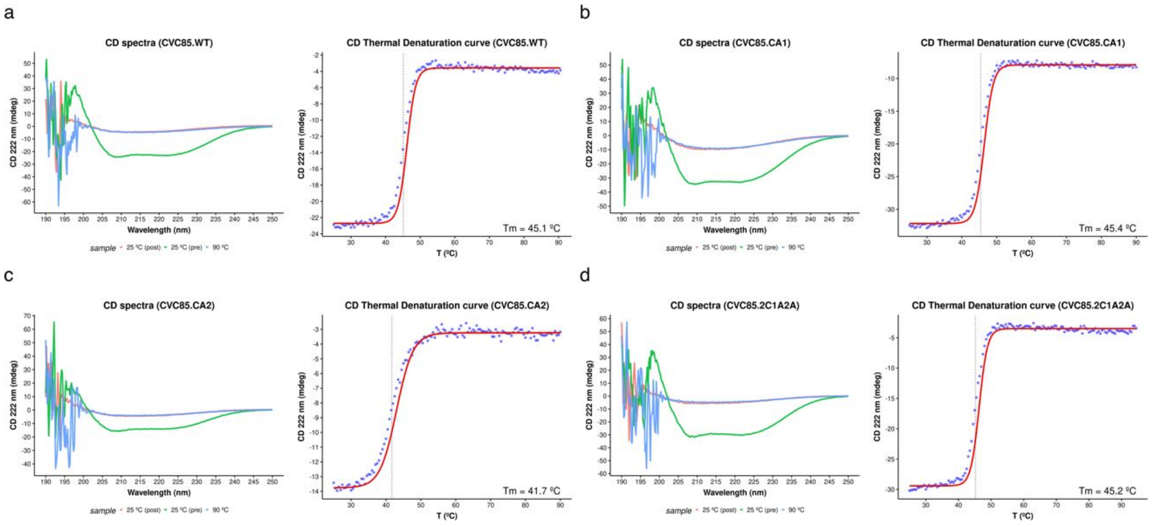

Supplementary Fig. 12. Folding and thermal stability of four CR3 αI mutants assessed by circular dichroism (CD). All mutant variants of the CR3 αI that could be expressed and purified in this study were subjected to CD to confirm the integrity of the fold at 25 ºC before and after thermal denaturation at 92.5 ºC, and their melting temperature was determined by following their thermal unfolding at 222 nm. **a**, WT: wild-type (T_m = 45.11 ± 0.04 ºC). **b**, 1A: R261A/R293A (T_m = 45.42 ± 0.03 ºC). **c**, 2A: R216/K231A (T_m = 41.69 ± 0.08 ºC). **d**, 1A2A: R261A/R293A/R216A/K231A (T_m = 43.49 ± 0.07 ºC). Source data are provided as a Source Data file.

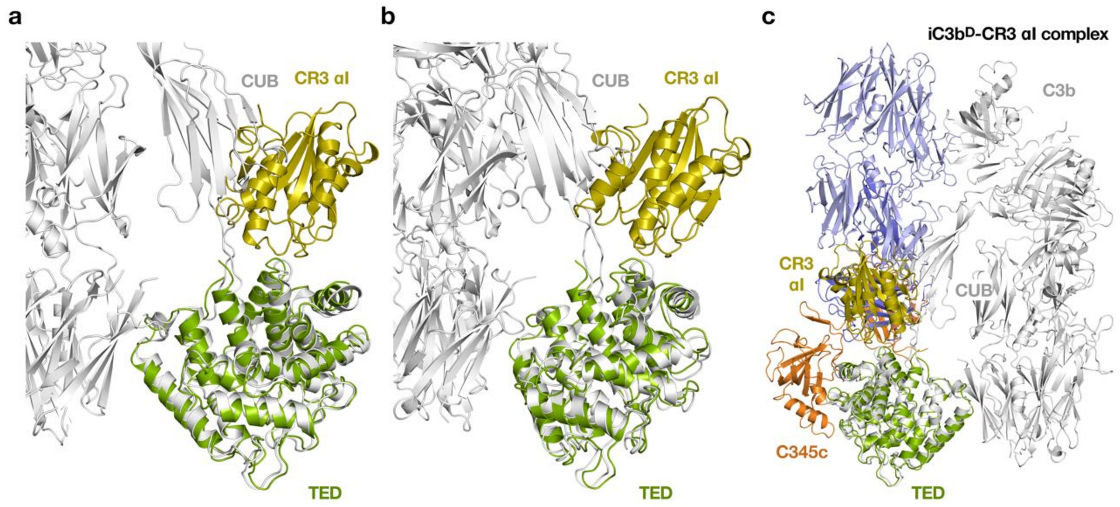

Supplementary Fig. 13. Structural basis of the negative selectivity of CR3 for C3b. a, Superposition of the TED-CR3 αI subcomplex of iC3b^{D}-CR3 αI complex onto C3b (PDB 2I07 [http://doi.org/10.2210/pdb2I07/pdb])[33]. **b**, Same superposition shown in (a) but rotated 30º around an in-plane vertical axis. (c) Overall view of the superposition including the entire iC3b^{D}-CR3 αI complex. Molecules are shown in cartoon representation and color coded as in the main text. C3b is shown in white. It is clear from the superposition that C3b does not admit the interaction with CR3 because of extensive clashes between the CUB domain and the loops connecting the CUB and the TED domains in C3b and the αI domain of CR3. Likewise, for the same reasons of steric hindrance it seems unfeasible that a CR3 molecule could simultaneously engage an iC3b molecule via the TED domain and a second C3b molecule through interactions with the MG6-MG7 domains.

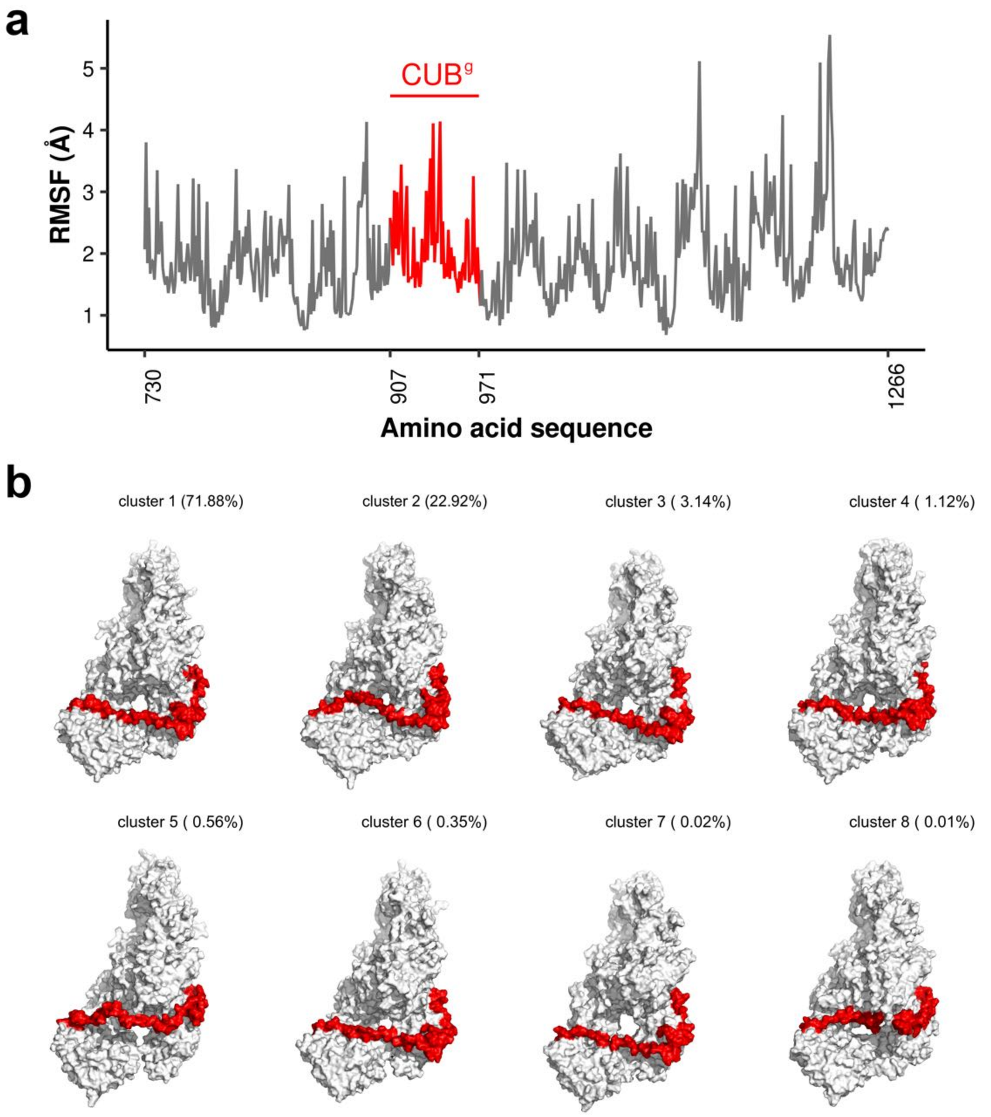

(Legend on the next page.)

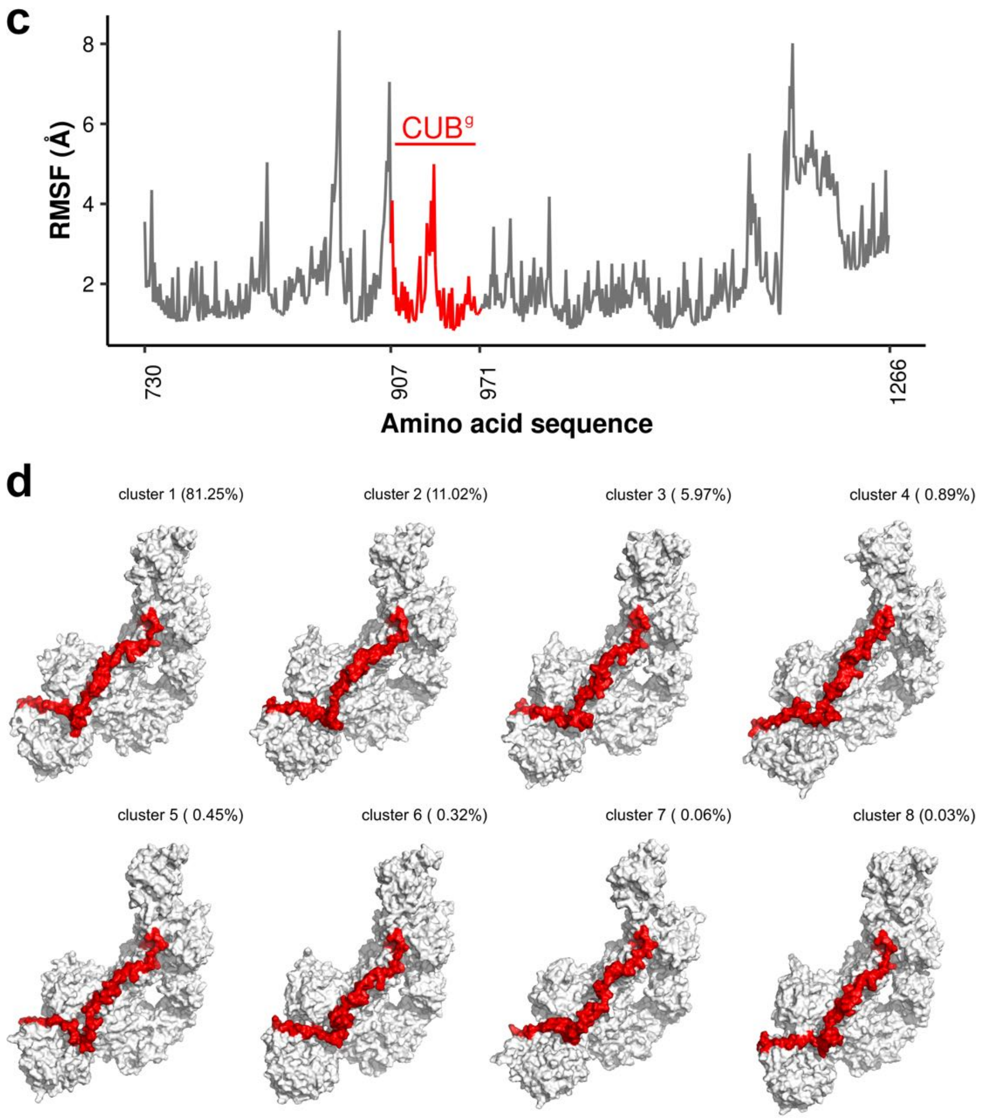

Supplementary Fig. 14. Molecular dynamics of iC3b-CR3 αI complexes modeled with the complete CUB^g motif. a, Plot of root-mean-square fluctuations (RMSF) *vs.* the indexed sequence of iC3b^D α63 chain encompassing a modeled CUB^g motif (residues 913−954), extracted from a 1000-ns molecular dynamics trajectory calculated for the iC3b^D-CR3 αI complex. RMSF values are expressed in Å. Segment 907−971 (which contains the CUB^g motif) is shown in red, whereas the rest of the RMSF values for the α63 chain is shown in black. **b**, Surface representation of the central structure of eight top-most clusters of the iC3b^D-CR3 αI complex, each one labeled with the cluster's frequency of occurrence. Segment 907−971 is in red. **c**, Like **a**

but for $iC3b^{U}$-CR3 α**I**. **d**, Like **b** but for $iC3b^{U}$-CR3 α**I**. Source data are provided as a Source Data file.

Supplementary References

1. Pernot, P. *et al.* Upgraded ESRF BM29 beamline for SAXS on macromolecules in solution. *J. Synchrotron Radiat.* **20**, 660–664 (2013).

2. Round, A. *et al.* BioSAXS Sample Changer: a robotic sample changer for rapid and reliable high-throughput X-ray solution scattering experiments. *Acta Crystallogr. D Biol. Crystallogr.* **71**, 67–75 (2015).

3. Franke, D. *et al.* ATSAS 2.8: a comprehensive data analysis suite for small-angle scattering from macromolecular solutions. *J. Appl. Crystallogr.* **50**, 1212–1225 (2017).

4. Hopkins, J. B., Gillilan, R. E. & Skou, S. BioXTAS RAW: improvements to a free open-source program for small-angle X-ray scattering data reduction and analysis. *J. Appl. Crystallogr.* **50**, 1545–1553 (2017).

5. Konarev, P. V. & Svergun, D. I. A posteriori determination of the useful data range for small-angle scattering experiments on dilute monodisperse systems. *IUCrJ* **2**, 352–360 (2015).

6. Svergun, D. I. Determination of the regularization parameter in indirect-transform methods using perceptual criteria. *J. Appl. Crystallogr.* **25**, 495–503 (1992).

7. Rambo, R. P. & Tainer, J. A. Accurate assessment of mass, models and resolution by small-angle scattering. *Nature* **496**, 477–481 (2013).

8. Petoukhov, M. V. *et al.* New developments in the ATSAS program package for small-angle scattering data analysis. *J. Appl. Crystallogr.* **45**, 342–350 (2012).

9. Petoukhov, M. V. & Svergun, D. I. Ambiguity assessment of small-angle scattering curves from monodisperse systems. *Acta Crystallogr. D Biol. Crystallogr.* **71**, 1051–1058 (2015).

10. Franke, D. & Svergun, D. I. DAMMIF , a program for rapid ab-initio shape determination in small-angle scattering. *J. Appl. Crystallogr.* **42**, 342–346 (2009).

11. Svergun, D. I. Restoring low resolution structure of biological macromolecules from solution scattering using simulated annealing. *Biophys. J.* **76**, 2879–2886 (1999).

12. Franke, D., Jeffries, C. M. & Svergun, D. I. Correlation Map, a goodness-of-fit test for one-dimensional X-ray scattering spectra. *Nat. Methods* **12**, 419–422 (2015).

13. Kozin, M. B. & Svergun, D. I. Automated matching of high- and low-resolution structural models. *J. Appl. Crystallogr.* **34**, 33–41 (2001).

14. Tuukkanen, A. T., Kleywegt, G. J. & Svergun, D. I. Resolution of ab initio shapes determined from small-angle scattering. *IUCrJ* **3**, 440–447 (2016).

15. Hess, B., Kutzner, C., van der Spoel, D. & Lindahl, E. GROMACS 4: Algorithms for Highly Efficient, Load-Balanced, and Scalable Molecular Simulation. *J. Chem. Theory Comput.* **4**, 435–447 (2008).

16. Emsley, P., Lohkamp, B., Scott, W. G. & Cowtan, K. Features and development of Coot. *Acta Crystallogr. D Biol. Crystallogr.* **66**, 486–501 (2010).

17. Williams, C. J. *et al.* MolProbity: More and better reference data for improved all-atom structure validation. *Protein Sci.* **27**, 293–315 (2018).

18. Jorgensen, W. L., Maxwell, D. S. & Tirado-Rives, J. Development and Testing of the OPLS All-Atom Force Field on Conformational Energetics and Properties of Organic Liquids. *J. Am. Chem. Soc.* **118**, 11225–11236 (1996).

19. Kaminski, G. A., Friesner, R. A., Tirado-Rives, J. & Jorgensen, W. L. Evaluation and Reparametrization of the OPLS-AA Force Field for Proteins via Comparison with Accurate Quantum Chemical Calculations on Peptides. *J. Phys. Chem. B* **105**, 6474–6487 (2001).

20. Jorgensen, W. L., Chandrasekhar, J., Madura, J. D., Impey, R. W. & Klein, M. L. Comparison of simple potential functions for simulating liquid water. *J. Chem. Phys.* **79**, 926 (1983).

21. Nosé, S. A molecular dynamics method for simulations in the canonical ensemble. *Mol. Phys.* **52**, 255–268 (1984).

22. Hoover, W. G. Canonical dynamics: Equilibrium phase-space distributions. *Phys. Rev. A Gen. Phys.* **31**, 1695–1697 (1985).

23. Parrinello, M. Polymorphic transitions in single crystals: A new molecular dynamics method. *J. Appl. Phys.* **52**, 7182 (1981).

24. Nosé, S. & Klein, M. L. Constant pressure molecular dynamics for molecular systems. *Mol. Phys.* **50**, 1055–1076 (1983).

25. Berendsen, H. J. C., Postma, J. P. M., van Gunsteren, W. F., DiNola, A. & Haak, J. R. Molecular dynamics with coupling to an external bath. *J. Chem. Phys.* **81**, 3684 (1984).

26. Darden, T., York, D. & Pedersen, L. Particle mesh Ewald: An N·log(N) method for Ewald sums in large systems. *J. Chem. Phys.* **98**, 10089 (1993).

27. Essmann, U. *et al.* A smooth particle mesh Ewald method. *J. Chem. Phys.* **103**, 8577 (1995).

28. Hess, B. P-LINCS: A Parallel Linear Constraint Solver for Molecular Simulation. *J. Chem. Theory Comput.* **4**, 116–122 (2008).

29. Miyamoto, S. & Kollman, P. A. Settle: An analytical version of the SHAKE and RATTLE algorithm for rigid water models. *J. Comput. Chem.* **13**, 952–962 (1992).

30. Daura, X. *et al.* Peptide Folding: When Simulation Meets Experiment. *Angew. Chem. Int. Ed.* **38**, 236–240 (1999).

31. Krissinel, E. Macromolecular complexes in crystals and solutions. *Acta Crystallogr. D Biol. Crystallogr.* **67**, 376–385 (2011).

32. Janssen, B. J. C. *et al.* Structures of complement component C3 provide insights into the function and evolution of immunity. *Nature* **437**, 505–511 (2005).

33. Janssen, B. J. C., Christodoulidou, A., McCarthy, A., Lambris, J. D. & Gros, P. Structure of C3b reveals conformational changes that underlie complement activity. *Nature* **444**, 213–216 (2006).

34. Krissinel, E. & Henrick, K. Secondary-structure matching (SSM), a new tool for fast protein structure alignment in three dimensions. *Acta Crystallogr. D Biol. Crystallogr.* **60**, 2256–2268 (2004).

35. Kabsch, W. A solution for the best rotation to relate two sets of vectors. *Acta Cryst. A* **32**, 922–923 (1976).

36. Bajic, G., Yatime, L., Sim, R. B., Vorup-Jensen, T. & Andersen, G. R. Structural insight on the recognition of surface-bound opsonins by the integrin I domain of complement receptor 3. *Proc Natl Acad Sci USA* **110**, 16426–16431 (2013).

37. Sen, M. & Springer, T. A. Leukocyte integrin $\alpha_L\beta_2$ headpiece structures: The αI domain, the pocket for the internal ligand, and concerted movements of its loops. *Proc Natl Acad Sci USA* **113**, 2940–2945 (2016).

38. Veevers, R. & Hayward, S. Methodological improvements for the analysis of domain movements in large biomolecular complexes. *Biophys. Physicobiol.* **16**, 328–336 (2019).
